# Prokaryotic Community Structure and Metabolisms in Shallow Subsurface of Atacama Desert Playas and Alluvial Fans After Heavy Rains: Repairing and Preparing for Next Dry Period

**DOI:** 10.3389/fmicb.2019.01641

**Published:** 2019-07-24

**Authors:** Miguel Ángel Fernández-Martínez, Rita dos Santos Severino, Mercedes Moreno-Paz, Ignacio Gallardo-Carreño, Yolanda Blanco, Kimberley Warren-Rhodes, Miriam García-Villadangos, Marta Ruiz-Bermejo, Albert Barberán, David Wettergreen, Nathalie Cabrol, Víctor Parro

**Affiliations:** ^1^Centro de Astrobiología (CAB, CSIC-INTA), Madrid, Spain; ^2^Carl Sagan Center, SETI Institute, Mountain View, CA, United States; ^3^NASA Ames Research Center, Moffett Field, Mountain View, CA, United States; ^4^Department of Soil, Water, and Environmental Science, University of Arizona, Tucson, AZ, United States; ^5^Carnegie Mellon University, Robotics Institute, Pittsburgh, PA, United States

**Keywords:** microbial ecology, Atacama Desert playa, rainfall event, subsurface environments, high-throughput DNA sequencing, metaproteomics, immunoassay microarrays

## Abstract

The Atacama Desert, the oldest and driest desert on Earth, displays significant rains only once per decade. To investigate how microbial communities take advantage of these sporadic wet events, we carried out a geomicrobiological study a few days after a heavy rain event in 2015. Different physicochemical and microbial community analyses were conducted on samples collected from playas and an alluvial fan from surface, 10, 20, 50, and 80 cm depth. Gravimetric moisture content peaks were measured in 10 and 20 cm depth samples (from 1.65 to 4.1% w/w maximum values) while, in general, main anions such as chloride, nitrate, and sulfate concentrations increased with depth, with maximum values of 13–1,125; 168–10,109; and 9,904–30,952 ppm, respectively. Small organic anions such as formate and acetate had maximum concentrations from 2.61 to 3.44 ppm and 6.73 to 28.75 ppm, respectively. Microbial diversity inferred from DNA analysis showed Actinobacteria and Alphaproteobacteria as the most abundant and widespread bacterial taxa among the samples, followed by Chloroflexi and Firmicutes at specific sites. Archaea were mainly dominated by Nitrososphaerales, Methanobacteria, with the detection of other groups such as Halobacteria. Metaproteomics showed a high and even distribution of proteins involved in primary metabolic processes such as energy production and biosynthetic pathways, and a limited but remarkable presence of proteins related to resistance to environmental stressors such as radiation, oxidation, or desiccation. The results indicated that extra humidity in the system allows the microbial community to repair, and prepare for the upcoming hyperarid period. Additionally, it supplies biomarkers to the medium whose preservation potential could be high under strong desiccation conditions and relevant for planetary exploration.

## Introduction

The Atacama Desert, located in northern Chile, is one of the driest and oldest hot deserts on Earth (Azua-Bustos et al., 2015; Bozkurt et al., 2016). Surrounded by the Coastal Range on the West and the Andes Mountains to the East, the Atacama Desert exhibits a diversity of polyextreme environments, showing arid to hyperarid conditions due to the influence of the stable Pacific Anticyclone (strengthened by the Humboldt Current) and the rain shadow effect of the Andean Cordillera (Houston and Hartley, 2003; Azua-Bustos et al., 2012; Bozkurt et al., 2016; Wierzchos et al., 2018). A paleohydrological study showed that the southern Atacama area displayed only three significant pluvial periods for the last 15 ka (Sáez et al., 2016). In this region, significant rainfall events (i.e. ≥2 mm, resulting in water movement and soil water activity >0.95, considered the limit for microbial life, Davis et al., 2010) are rare, typically occurring only once per decade (Houston, 2006; Robinson et al., 2015; Sáez et al., 2016). The long evolution of the Atacama Desert from a semi-arid climate in the Jurassic (*c.* 200 million years ago) to extreme hyperaridity – that arose in the Miocene, *c.* 15 million years ago – (Dunai et al., 2005; Hartley et al., 2005; Clarke, 2006; Gómez-Silva et al., 2008), coupled with high diurnal-nocturnal thermal fluctuations, intense ultraviolet (UV) radiation and the presence of specific inorganic compounds such as chlorides, perchlorates and halite in soils, make this desert one of the most relevant terrestrial analogues of Mars (Chong-Díaz et al., 1999; Parro et al., 2011; Bull and Asenjo, 2013; Bull et al., 2016; Wilhelm et al., 2018).

Despite severe desiccation, high salinity, and UV radiation, different microorganisms are able to survive in the Atacama Desert, either on the soil surface or within/under rocks and salts (Warren-Rhodes et al., 2006; Bull and Asenjo, 2013; Crits-Christoph et al., 2013; Davila et al., 2015; Schulze-Makuch et al., 2018; Wierzchos et al., 2018), or even in temporary hypersaline lagoons (Azua-Bustos et al., 2018). All these environments to date, however, have been shown to harbor exceedingly low microbial abundance and biomass (Crits-Christoph et al., 2013; Schulze-Makuch et al., 2018; Warren-Rhodes et al., 2019). Atacama subsurface soils also present hyperarid conditions together with a high presence of chloride-bearing salts, thus creating a favorable environment for better preservation of a wide range of molecular biomarkers through a process called xeropreservation: a taphonomic process in which biomarkers are preserved by drying as low water activity greatly reduces both biological and chemical degradation (Fish et al., 2002; Stan-Lotter et al., 2006; Rethemeyer et al., 2010; Parro et al., 2011; Wilhelm et al., 2017). However, microbial ecology studies investigating these subsurface environments have been limited.

Additionally, due to the xeropreservation process typical of Atacama subsurface environments, a highly varied protein biomarkers stock from previously active microorganisms is expected in these areas. The identification of proteins and enzymes provides reliable information about the microbial functionality and stress-response mechanisms in soils, as well as the potential role of microbial communities in major biogeochemical cycles – e.g., carbon, nitrogen or phosphorus (Maron et al., 2007; VerBerkmoes et al., 2009; Schneider and Riedel, 2010; Bastida et al., 2014). Nonetheless, functional soil microbial ecology studies are quite scarce and have only recently been undertaken (Bull et al., 2016; Schulze-Makuch et al., 2018; Uritskiy et al., 2018), thus making soil metaproteomics description a general challenge for microbial ecologists (Bastida et al., 2014).

Within different soil ecosystems in the Atacama Desert, “playa” soils represent orographically and environmentally remarkable features, covering extensive areas that deserve further study. A “playa” is a flat-bottom basin with a negative water balance for most of the time and for the annual total, located in endorheic topographic low areas and characterized by highly distinct environmental composition and dynamics both above and below their surfaces (Rosen, 1994; Briere, 2000; Navarro et al., 2009; Shaw and Bryant, 2011; Kidron, 2016; Goldstein et al., 2017). Soil surfaces of these playas, with low organic matter and commonly lacking biocrusts, are mainly composed of low-porosity fine-grained soil particles cemented by salts (Bowler et al., 1986; Davis et al., 2010; Hang et al., 2015; Goldstein et al., 2017; McKenna and Sala, 2018). In these areas, water (almost exclusively from rainfall events) can rapidly penetrate into the subsurface, where it is less subjected to evapotranspiration processes and can persist longer, even recharging ground water (Reynolds et al., 2007; Hang et al., 2015; McKenna and Sala, 2018). Thus, playa surfaces rapidly dry out, becoming highly vulnerable to wind erosion (Kidron, 2016; Goldstein et al., 2017), in comparison to playa subsurfaces, which show enhanced water-retention capacity, thus presenting favorable protected (albeit temporary) habitable microenvironments for community survival and growth.

This study aims to investigate the effect of sporadic wet events on surface and subsurface microbial communities and biomarker content in the widespread playa ecosystems in the driest core of the Atacama Desert. Samples were collected in March 2015, 3–5 days after an unprecedented heavy rain that accumulated 40–90 mm within the core of the Atacama Desert, where precipitation usually totals only a few to tens of mm a year (Bozkurt et al., 2016; Dirección meteorológica de Chile, 2018). Although different recent studies (e.g., Schulze-Makuch et al., 2018; Uritskiy et al., 2018) have focused on several hyperarid sites of the Atacama Desert after the same 2015 rainfall event, none focused on playas, nor were completed within days of the event. By using geochemical, immunological, DNA sequencing, and metaproteomic techniques, we determined the variable physicochemical parameters and the associated microbial community structure and metabolisms along different vertical profiles.

## Materials and Methods

### Sampling and Site Description

**Figure 1:**
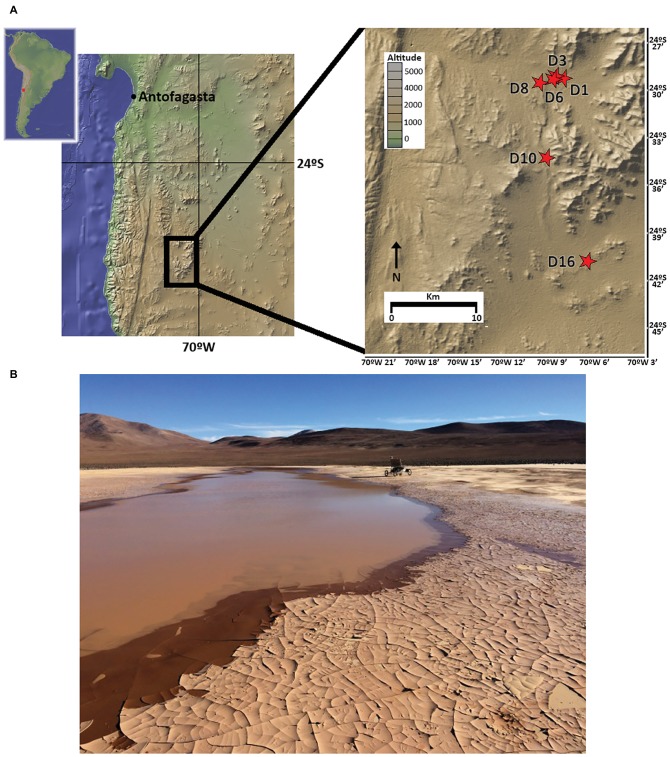
Sampling locations in the Atacama Desert, Chile. **(A)** Map of the sampling sites (red stars). **(B)** Image of northernmost playa site (sampling points D1, D3, and D6) 3 days after rainfall, with rover Zoe in the background (photography by David Wettergreen, used with permission).

As part of the LITA project rover field plan on 2015, five sample sites in playa soils (D1, D3, D6, D10, and D16) and one in an alluvial fan (D8) site located in the Atacama Desert were selected to cover a longitudinal (West-East) transect (Figure 1). Up to 3 kg of soil samples were independently and aseptically collected in Whirl-Pak^®^ bags 3–5 days following the 2015 rainfall event that delivered 42.5 mm to the study site within an ~24 h period (recorded at ESO Paranal Observatory, *c.* 40 km from the study site). Samples covered a vertical profile, from surface (hereafter, SF), 10, 20, 50, and 80 cm depths at each sampling site. A small fraction of each sample was utilized for *in situ* field experiments; remaining soils were immediately frozen at −20°C for shipment and storage until laboratory analysis.

### Geochemical and Mineralogical Analyses

The analyses of a selected group of inorganic and organic anions were carried out by ion chromatography (hereafter IC) using 2 g of sample dissolved in 40 ml of distilled water for IC (Sigma-Aldrich, ref. no. 00612), with agitation for 2 days. Samples were then centrifuged at 4000 RCF to remove mineral particles. Supernatants were collected and loaded into a Metrohm 861 Advanced Compact Ion Chromatographer IC (Metrohm AG, Herisau, Switzerland), undiluted or diluted at either 50 or 20%. All the anions were eluted with 3.6 mM sodium carbonate (NaCO_3_) through the column *Metrosep A supp 7-250*. Calibration curves were performed, either for a set of compounds (fluoride, chloride, bromide, nitrite, nitrate, phosphate, sulfate, acetate, formate, propionate, tartrate, and oxalate) separately and in mixtures. Only those sample values that fit in the linear part of the calibration curves were considered. The pH of these same water solutions was measured with an inoLab pH meter (WTW, GmbH & Co. KG, Weilheim, Germany) after 24 h of solution stabilization.

The identification of monosaccharides and related compounds was carried out on D3, D6, and D8 samples by means of GC-MS, following protocols in Blanco et al. (2019). Further details can be found in Supporting Information S1.

Powder X-Ray Diffraction (hereafter, XRD) was performed using a Seifert 3003 TT with Cu Kα radiation (*λ* = 1.542 Å). The X-ray generator acceleration voltage was 40 kV, with a filament emission of 40 mA. A step size of 0.1° (2*θ*) and a count time of 2 s were used to scan the samples between 5° (2*θ*) and 60° (2*θ*).

Gravimetric moisture content analyses of the samples were carried out with 2 g of soil that were weighed before and during (24 h) drying at 110°C until the weight stabilized (normally after 48 h).

### DNA Extraction, PCR Amplification and Sequencing

Genomic DNA was extracted from D3, D6, and D8 soil samples independently using the PowerMax Soil DNA Isolation Kit (MO BIO Laboratories, INC.) with slight variations to standard procedures in the lysis step: samples were incubated with lysis solution (“Solution C1” in the kit) for 1 h at room temperature and then horizontally vortexed for 20 min. All extraction reagents and reaction tubes were previously sterilized with UV-C radiation (30 mV, 5 min) using a GS gene linker UV chamber (Bio-Rad laboratories, Hercules, CA, USA) for reducing possible kit reagent contamination. A control extraction sample was carried out following all kit steps but with no soil sample included to look for possible resistant kit reagent contamination. Extracted DNA solutions were concentrated using a Speed-Vac concentrator (Savant Inc.) and purified using the QiaEX II DNA Purification Kit (Qiagen Laboratories, INC.). Concentrations from samples and controls were then determined in a NanoDrop ND 1000 spectrophotometer (Thermo Fisher Scientific TM).

Soil bacterial and archaeal communities were identified at three different depths (surface, 20 and 80 cm depth) by construction of paired-end amplicon library by means of Illumina MiSeq sequencing (Illumina Inc., San Diego, CA, USA). Bacterial *16S* rRNA V3-V4 gene region was amplified using the primer pair 341-F/805-R (Herlemann et al., 2011), while archaeal *16S* rRNA V2-V3 region was amplified using the primer pair Arch1F/Arch1R (Cruaud et al., 2014). PCRs and Illumina MiSeq sequencing were carried out at the NGS-Genomic Service of the Fundación Parque Científico de Madrid (FPCM) according to their protocols (Supporting Information S2). DNA control extraction sample, obtained following all kit steps but with no soil sample included, did not amplify during PCR steps, so it was not included into the sequencing process. Control amplifications (solutions of all PCR reagents but with sterile distilled water instead of DNA sample) were also carried out at the genomic service laboratory to look for possible PCR reagents contamination, without yielding any positive result. Raw sequence data were deposited at the NCBI Sequence Read Archive (SRA, http://www.ncbi.nlm.nih.gov/sra) under accession number PRJNA506644.

### Sequence Processing and Analysis

Raw sequences from bacterial and archaeal *16S* rRNA regions were processed in MOTHUR software v.1.39.5 (Schloss et al., 2009), using a custom script based upon MiSeq SOP (Kozich et al., 2013). Briefly, reads containing below a minimum number of bp (≤400 bp for bacteria and ≤300 bp for archaea), those containing ambiguous nucleotide identities (“Ns”) and/or homopolymers longer than 8 bp, as well as singletons and/or those identified as putatively chimeric, were removed from subsequent analyses. Remaining sequence reads were then clustered into Operational Taxonomic Units (OTUs) at the 97% similarity level. Datasets were rarefied independently to even sequencing depth, corresponding to the lesser number of sequences in the samples (95,756 for bacteria, 1,637 for archaea). Sequencing depth for each sample was tested by means of rarefaction curves constructed using iNEXT Online (Chao et al., 2016) (Supporting Information S12). Taxonomic affinities for the reads were assigned by comparison of OTUs representative sequences against RDP database (RDP v.16 reference files; release 11, Cole et al., 2013). OTU’s affinities reported as “cyanobacteria/chloroplast” were further assigned a taxonomic identity by comparing them against nr/nt (NBCI), EMBL, Greengenes and SILVA databases for more precise cyanobacterial taxonomic identification. Sequences assigned to “mitochondria” or chloroplast were removed from further analyses. Bacterial and archaeal OTU richness (*S*) and Shannon diversity index (*H*′) were calculated per sample using the R package “vegan” v.2.4-3 (Oksanen, 2017).

### Metaproteomics

Protein extracts were prepared from 40 g of soil with GuHCl buffer (4 M guanidine-hydrochloride, 0.5 M EDTA, 0.5 M Tris, pH 7.6), following Tuross and Stathoplos (1993). Purification and concentration of extracted proteins were carried out using up to 300 μg of protein extract and subsequently subjected to direct trypsin digestion.

Peptide identification from raw data was carried out using licensed search engine MASCOT v.2.3.0^1^. Peptides were assigned an identity by a search performed against Swissprot database without taxonomic restriction (date 08/02/16; 550,116 sequences). Those proteins identified by only one peptide present in one unique sample were removed from the dataset prior to subsequent analyses. Detailed information on metaproteomic analyses are shown in Supporting Information S3.

Protein identifiers (protein IDs) coming from Swissprot database were used to retrieve amino acid complete fasta sequences from Uniprot web server (Uniprot consortium, 2017), using the Retrieve/IDmapping tool (“from UniprotKB AC/ID to UniprotKB” option). These sequences were then used as input for Blast2GO software (Conesa and Götz, 2008). This software was used to further assign proteins to Gene Ontology (hereafter, GO) terms as well as to enzyme codes and KEGG pathways following standard software options.

### Sandwich Microarray Immunoassays With LDChip200

Samples were analyzed by fluorescent sandwich microarray immunoassays (FSMI) using the LDChip200 (i.e. Life Detector Chip; Parro et al., 2008, 2011; Sánchez-García et al., 2018). LDChip200 is an antibody microarray-based biosensor which contains about 200 polyclonal antibodies (purified IgG fraction) designed to identify an assortment of biological polymers (including lipo/exo-polysaccharides), conserved proteins and peptides involved in key metabolisms (e.g. nitrogen fixation, nitrogen and sulfur reduction, energy metabolisms, or iron homeostasis), bacteria and archaea belonging to main taxonomic groups (including spores from Gram-positive bacteria) and crude environmental extracts from extreme environments (Rivas et al., 2008; Parro et al., 2011). The targets for the antibodies of this study are described in Sánchez-García et al. (2018). IgG fraction of each antibody was printed in a triplicate spot-pattern on the surface of epoxy-activated glass slides as described in Blanco et al. (2017). To perform the FSMI, all IgGs were fluorescently labeled with Alexa 647, titrated, and used in a mixture that consisted of 181 antibodies to reveal the immunoreactions as reported by Rivas et al. (2008).

Detailed protocol for the analysis of LITA samples with the LDChip200 is described in Blanco et al. (2017). In summary, 0.5 g of each protein extract (see previous section) was resuspended in 2 ml of TBSTRR buffer (0.4 M Tris-HCl pH 8, 0.3 M NaCl, 0.1% Tween 20) and was used as a multianalyte-containing sample for the FSMI. The LDChip200 images were analyzed and quantified by GenePix Pro Software (Molecular Devices, Sunnyvale, CA, USA). The final fluorescence intensity (*F*) was quantified as previously reported (Rivas et al., 2011; Blanco et al., 2012). An additional cutoff value, which was the first interval of *F* with an accumulated frequency higher than 80% and an increase less than 10% from the previous one, was applied to minimize the number of false positives. Output fluorescence data were normalized prior to downstream analysis attending to number of probes per taxonomic/metabolic group and to total microarray fluorescence values following He et al. (2007) protocols. In order to correlate LDChip and proteomics results, Blast2GO software (Conesa and Götz, 2008) was used for custom BLAST searches of peptides sequences detected in LDChip against a database containing all proteins and inferred peptide sequences from metaproteomics.

### Statistical Analysis

Bray-Curtis distances were calculated using the R package “vegan” v.2.4-3 (Oksanen, 2017) for physicochemical factors on log-normalized data, on the abundance of microbial OTUs, and on the presence of individually identified protein data coming from metaproteomic sequencing analyses. These three distance matrices were visualized using non-metric multidimensional scaling (NMDS) with R package “vegan” v.2.4-3. Permutational ANOVA (PerMANOVA), based on these same Bray-Curtis distances and using 999 permutations, and were employed to test the effect of sampling site, depth, and environment (playa vs. alluvial fan) – as fixed variables – and of their interactions in microbial community composition (OTU-based) and protein identification with R package “vegan” v.2.4-3. Conditional independence relationships between bacterial classes or major KEGG pathways and the different samples were assessed by Correspondence Analysis (CA) carried out also in SPSS v.24.0 software. Venn diagrams of shared and unique proteins were constructed by means of Venny v.2.1 online tool (Oliveros, 2015).

## Results

### Physicochemical Parameters and Their Vertical Patterns in the Atacama Playas

To investigate surface and subsurface mineralogical and geochemical context, samples were analyzed by X-Ray diffraction (XRD) and ion chromatography (IC) techniques. XRD analyses showed a widespread presence of phyllosilicates and tectosilicates, with quartz and albite in most of the samples and depths, and microcline irregularly distributed among the samples (Supporting Information S4). Gypsum was only detected for deeper samples in D1 and D8 sites. Other detected minerals such as minor phyllosilicates and silicates (olivine, mica, muscovite, annite, takanelite, pargasite, clinochlore, zinnwaldite, and katayamalite), phosphates (scorzalite), iron minerals (hematite and orthopyroxene) or other metal-containing ones (billietite and kutnahorite) were present in one or a few samples, but did not show any recognizable trend.

**Figure 2:**
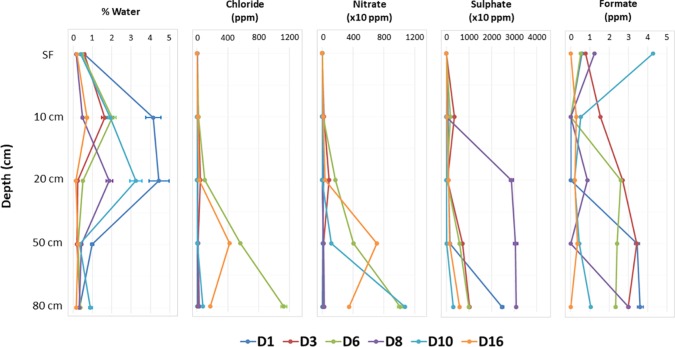
Variations with depth of different physicochemical factors in the sampling points. Note the different scale in horizontal axis for nitrate and sulfate concentration.

Geochemical analyses by IC (Supporting Information S5) allowed the detection of the most soluble organic and inorganic anions, which demonstrated some patterns with depth. The concentrations of chloride (maximum of 1125.07 ± 37.521 ppm in D6-80), nitrate (10767.30 ± 11.368 ppm in D10-80), and sulfate (30952.21 ± 36.245 ppm in D8-80) clearly increased with depth at most sites, as it did for formate (Figure 2, Supporting Information S5). Gravimetric moisture content (% wt/wt of water) showed a slight but significant peak between 10 and 20 cm for most sites (Figure 2, Supporting Information S5). Variable pH values were also observed among the different sampling points and depths, from 6.77 ± 0.675 in D8-SF to 8.94 ± 0.897 in D10-20, with the lowest values always found in SF samples, except for D10 and D16 (Supporting Information S5).

Samples presented different compositions of sugars, alcohols, and fatty acids, especially between playas and alluvial fan (Supporting Information S6). Glucose was broadly distributed among sites and depths, while galactose, mannose, and sorbitol were only detected in playa samples (D3 and D6), and palmitic acid was mainly found along D3 samples. Myoinositol was restricted only to D3-20, D3-80, and D6-10 samples.

Non-metric multidimensional scaling (NMDS) of all samples according to physicochemical parameters (Supplementary Figure S7) illustrated two clearly differentiated clusters: one comprising the top samples (all surficial and some from 10 and 20 cm depths) on the left of the ordination, and a second one comprising the deeper samples (rest of 10 and 20 cm plus all 50 and 80 cm depths) on the right of the ordination. In turn, this second group clearly showed a trend to segregate into two subclusters, one with 10 and 20 cm depth samples and the other with 50 and 80 cm ones. Water content, pH, protein, and sugar concentrations mapped between the surficial group and the 10–20 cm subcluster, being closer to the latter group. Most deep subcluster samples showed a marked correlation with sulfate, chloride, and nitrate concentrations.

### Microbial Community Diversity and Structure

**Figure 3:**
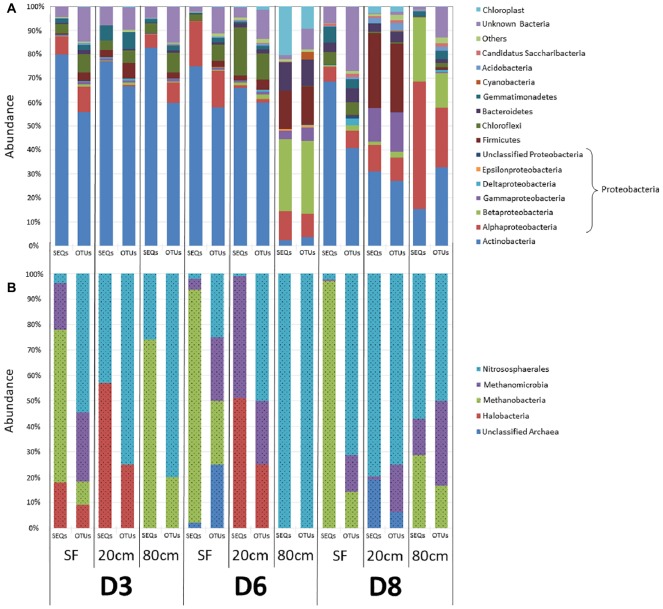
Distribution of prokaryotic communities among sampling points and depths. OTU richness (OTUs) and relative sequences abundances (SEQs) are expressed as a percentage of the total number of OTUs and sequence reads for each sample, respectively. **(A)** Bacterial phyla and classes. “Others” refer to taxa that account for <1% of relative sequence reads abundance within all samples. **(B)** Archaea classes. In both **(A)** and **(B)**, color legend order reflects the organization of data in bars, with increasingly abundant (on average) identified groups sited closer to the lower part of each bar.

To investigate microbial community composition from different habitats, we focused on three sites: two from the same playa with different humidity patterns (D3 and D6) and one from an alluvial fan (D8) 2 km distant from the playa (Figure 1). Total DNA was extracted and the prokaryotic (bacteria and archaea) *16S* rRNA gene was sequenced and analyzed. Results showed only minimal differences in the distribution of bacterial lineages for most playa samples, with the exception of D6-80, and of alluvial fan D8-20 and D8-80 sites (Figure 3A, Supporting Information S8). *Actinobacteria* populations were most abundant for the majority of the samples, with the exceptions of D6-80, D8-20, and D8-80. *Alphaproteobacteria* class was the second most represented and widely distributed taxonomic group, with the highest abundance in the D8-80 sample. *Chloroflexi* were also broadly distributed in the samples, but virtually absent from D6-80, D8-20, and D8-80. *Betaproteobacteria* were very abundant in D6-80 and D8-80, as well as *Gammaproteobacteria* and *Firmicutes* in D6-80 and D8-20, while *Cyanobacteria* presence was only comparatively significant in D6-80. Despite the even distribution of bacterial lineages in playa sites, statistically supported associations were observed between some lineages and samples by means of Correspondence Analysis (Supporting Information S9). *Alphaproteobacteria* and *Betaproteobacteria* classes clustered close to D8-80, while *Bacteroidetes*, *Cyanobacteria*, and *Epsilonproteobacteria* were associated with D6-80, and *Acidobacteria*, *Firmicutes*, and *Gammaproteobacteria* were found to cluster close to D8-20.

Within *Actinobacteria*, *Geodermatophilaceae* (27.8% of sequences, *Geodermatophilales* order), and *Actinomycetaceae* (15.4%, *Actinomycetales*) were the most abundant families, with the former mainly found in D3 samples and the latter showing a more widespread distribution. Within *Proteobacteria*, *Erythrobacteraceae* and *Sphingomonadaceae* (19.5 and 16% of sequences, respectively, both included in *Sphingomonadales* order), *Rhodobacteraceae* (19%, *Rhodobacterales*) and *Acetobacteraceae* (16.8%, *Rhodospirales*) were the most abundant families within *Alphaproteobacteria* class, being far more prevalent in D8-20 and D8-80. *Comamonadaceae* and *Burkholderiaceae* (*Burkholderiales* order) together accounted for more than 75% of *Betaproteobacteria* sequences, while *Moraxellaceae* (37.7% of sequences, *Pseudomonadales* order), *Xanthomonadaceae* (20.9%, Xanthomonadales) and *Pasteurellaceae* (17.5%, Pasteurellales) were the most represented families within *Gammaproteobacteria*.

DNA sequencing of archaeal *16S* rRNA showed only OTUs belonging to *Euryarchaeota* (*Haloarchaea*, *Methanobacteria*, and *Methanomicrobia* classes) and to *Thaumarchaeota* (*Nitrososphaera* class) (Figure 3B, Supporting Information S8). Surface samples (D3-SF, D6-SF and D8-SF), as well as D3-80, were dominated by *Methanobacteria*, while deeper samples had the highest abundance of *Haloarchaea*, *Methanomicrobia* and, especially, *Nitrososphaera*. It is also worth noting, in contrast to the above playa samples, the lack of detected haloarchaeal OTUs in D8 sampling points (Figure 3B, Supporting Information S8).

Bacterial OTU richness (*S*) in the samples ranged from 447 OTUs in D6-80 to 1,334 in D3-SF, albeit with no recognizable trend (Supporting Information S10A). Similarly, Shannon diversity index (*H′*) did not show any discernible patterns among samples, ranging from 2.85 in D8-80 to 4.28 in D8-20, although values were slightly higher at 20 cm depth on average (Supporting Information S10B). Archaeal OTU richness (*S*) in the samples ranged from 2 OTUs in D6-80 to 16 in D8-20, without presenting any recognizable trend (Supporting Information S11). Shannon diversity index (*H′*) for archaeal communities had much lower values than bacterial ones, ranging from 0.18 in D8-SF to 1.37 in D8-80 sample and did not show any significant relation to any environmental parameter (data not shown).

**Figure 4:**
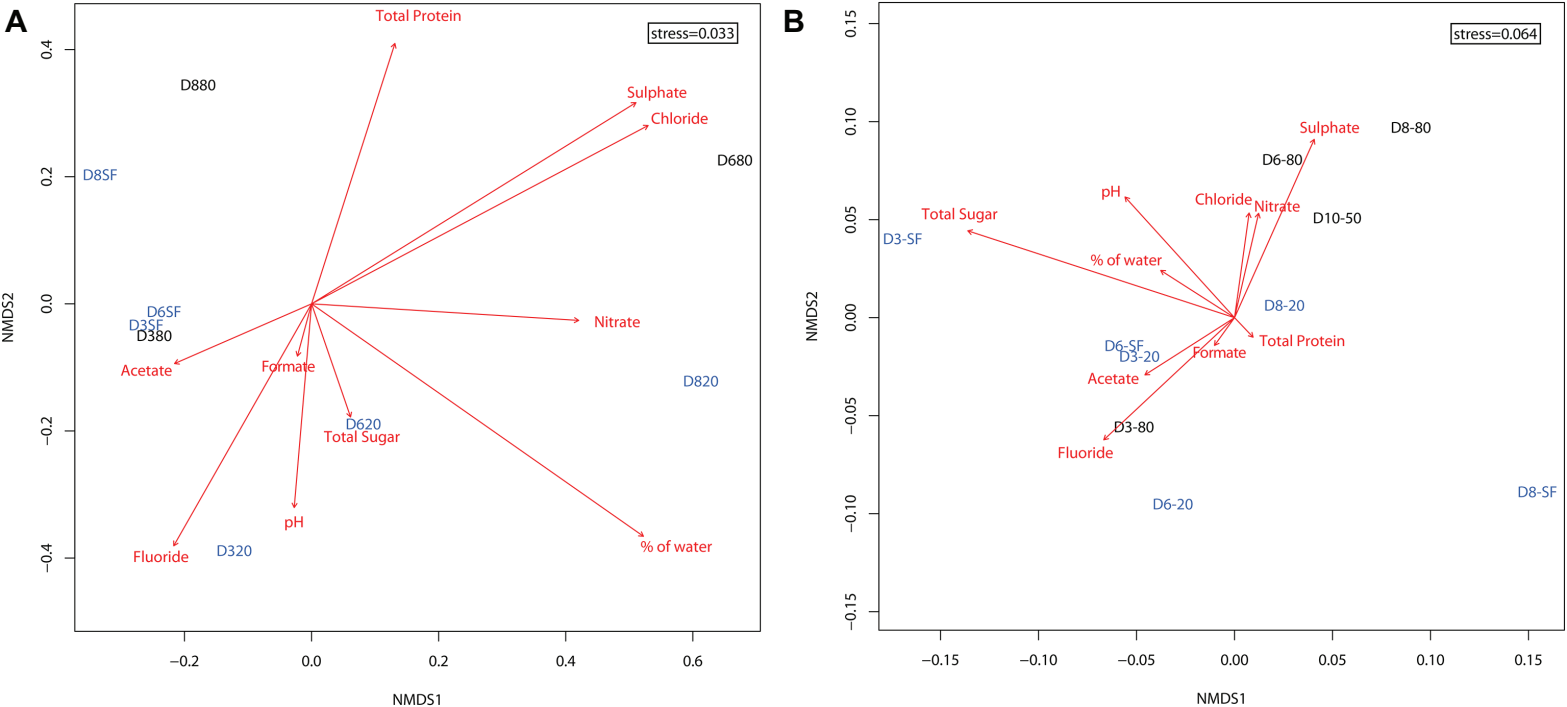
Two dimensional non-metric multidimensional scaling (NMDS) ordination plot of sampling points (based on Bray-Curtis similarities) for the distribution of **(A)** microbial OTUs (stress = 0.033) and **(B)** presence of proteins (stress = 0.064) in the samples. Shallow and intermediate depth samples (from surface – SF – and 20 cm depth, respectively) are shown in light blue. Deeper samples (from 50 and 80 cm depth) are shown in black. Red arrows represent the fitness of physicochemical factors into the ordination, with higher magnitude arrows corresponding to higher correlations to the data (arrow lengths cannot be compared across plots). Nitrite and bromide data were excluded from analyses due to their very scarce detection in the samples.

Ordination of microbial community compositions showed a clear association of samples with depth and, in a lesser extent, with the type of environment – playa or alluvial fan – (Figure 4A). Shallow samples (D3-SF, D6-SF, and slightly further D8-SF) clustered with D3-80. Twenty-centimeter depth samples at the three locations (D3-20, D6-20, and D8-20) were sited along the bottom of the ordination, with D3-20 and D6-20 clustering closer to each other than D8-20. According to their direction and magnitude, fluoride, pH, % water and nitrate were the most important factors driving microbial community composition at this depth. The deepest samples, D6-80 and D8-80, were located on the top of the ordination, with detected microorganisms clearly influenced by sulfate and chloride. These differences were statistically supported by PerMANOVA results, as they show the interaction of depth and type of environment as the only significant predictors of the differentiation of bacterial communities (*r*^2^ = 0.26, *p* < 0.05).

### Metaproteomic Study and Metabolic Pathways

Total protein content was extracted from sampling sites D3, D6, D8, and D10 and subjected to metaproteomic analysis. Ordination of samples considering only the presence or absence of proteins showed the deepest samples (D6-80, D8-80, and D10-50, with the exception of D3-80) clustering mainly with sulfate, chloride, and nitrate concentrations (Figure 4B). Twenty cm-depth samples, along with D3-80, formed their own cluster in the center of the ordination, mostly correlated with fluoride, acetate, and formate concentrations. Sample D3-SF seemed to be more related to total sugar concentration. Taking into account axis NMDS1, alluvial fan samples (D8) were sited at the right half of the axis, opposite to most playa ones (with the exception of deep D6-80 and D10-50). PerMANOVA results on these data support this segregation as they showed that only the effect of type of environment (playa or alluvial fan) was significantly influencing the presence of distinct microbial proteins (*r*^2^ = 0.26, *p* < 0.05).

**Figure 5:**
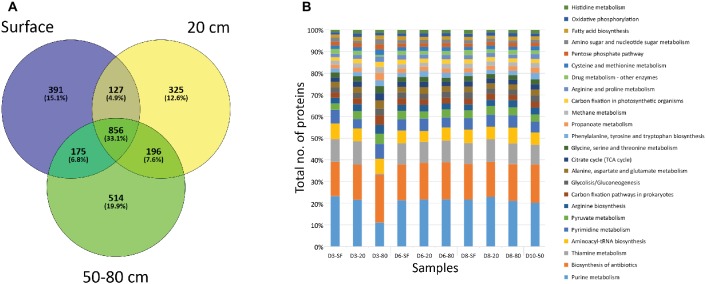
Distribution of proteins by depth of sampling. **(A)** Venn diagram showing the number of unique (i.e. only found at one depth) and shared proteins among soil samples grouped by depth. “SF” group comprises D3-SF, D6-SF, and D8-SF samples; “20 cm” group comprises D3-20, D6-20, and D8-20 samples; “50–80 cm” group comprises D3-80, D6-80, D8-80, and D10-50 samples. **(B)** Bar plot showing the distribution among sampling points of proteins related to different cellular processes, according to KEGG database project (see legend). Note that detected processes that account for less than 1% of the total number of proteins were not plotted.

A very high number of proteins (856, corresponding to the 33.1% of the total number of identified proteins) were shared among sampled depths (Figure 5A). However, unique proteins to each particular depth were also abundant: 391 (15.1% of total number proteins) to surface (SF) samples, 325 (12.6%) in 20 cm depth a,nd 514 (19.9%) for deep samples (50 and 80 cm depth) (Figure 5A). Despite being unique to each depth, most of these proteins were related to the same cellular biosynthetic metabolisms (by means of KEGG protein adscriptions, Figure 5B), such as DNA, RNA, and amino acid/protein synthesis (e.g. purine metabolism, aminoacyl-tRNA biosynthesis, pyrimidine metabolism, or arginine biosynthesis), and several biological processes (Supporting Information S13). Along with these cellular processes, a high number of proteins were mainly involved in energetic pathways, such as pyruvate metabolism or the tricarboxylic acid cycle (citrate/TCA cycle in Figure 5B). Additionally, proteins detected included those principally involved in autotrophic (carbon fixation, mainly carboxykinases, carboxylases, and dehydrogenases), chemolithotrophic (methane metabolisms, mostly transferases, such as thiotransferases and hydroxymethyltransferases), nitrogen cycling (mainly ammonia assimilation by means of ammonia ligases and other ligases and kinases), and sulfate reduction (i.e. sulfate adenylyltransferases – cysC, cysD, cysH, cysI, cysJ, cysN), as well as other proteins involved in biosynthesis pathways for sugars (especially glucose and galactose, as well as the glycolysis/gluconeogenesis pathway, Figure 5B), alcohols (myo-inostol) and fatty acids. Although these are the main metabolic activities in which the referred proteins take part, it is worth noting that some of them could bind more than one substrate, even presenting a reversible chemical behavior within the same cycles. Additionally, a relatively high number of proteins associated with ecological interactions such as biosynthesis of antibiotics and drugs or thiamine metabolism were detected. The even distribution of these activity biomarkers was statistically supported by a Correspondence Analysis (CA) not showing any specific association between them and any sampling point (data not shown).

Proteins involved in stress responses (identified by GO terms) were also detected, but in much lower quantities (altogether 1.76% of total identified proteins). However, a number of abundant proteins, identified by GO terms to be involved in different pathways, can also have a stress response function. This is the case for detected iron binding proteins (e.g. oxidative stress), nucleotide binding proteins (e.g. heat shock, nutrient scarcity, hyperosmotic stress) or cell division proteins (e.g. *sporulation*). Similarly to metabolic cellular processes, stress response proteins showed a homogenous distribution in sampling points and depths (Supporting Information S14). Of these, proteins involved in responses to oxidative stress and heat were again the most abundant, with an important incidence of response to osmotic and salt stresses pathways.

### Biomarker Profiling With a Life Detector Chip Multiplex Immunoassay

**Figure 6:**
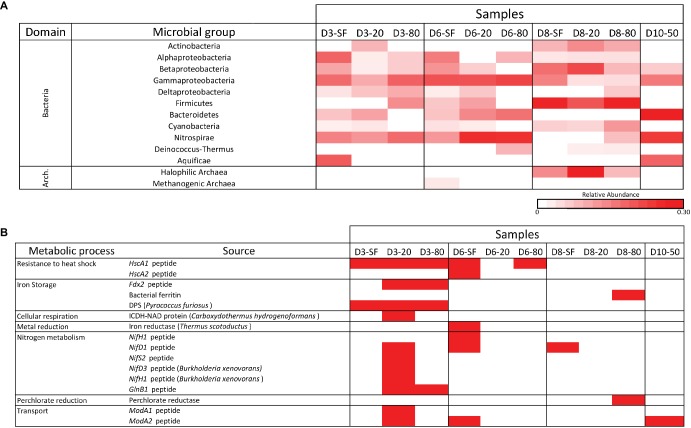
LDChip results distribution heatmaps. **(A)** Bacterial and archaeal groups among sampling points and depths (SF-surface, 20, 50, and 80 cm) by means of LDChip200 analyses. The antibodies recognized by LDChip200 (further information in Sánchez-García et al., 2018) were reorganized on the basis of the phylogeny of the targets. Average signal intensity corresponding to positive antibody reactions within each category was used as a proxy for relative abundance, calculated according to He et al. (2007). White color indicates non-detected signal, while intensity of positive signals is indicated from light pink (lower signal) to red (higher signal). **(B)** Positive immunosignals related to microbial metabolism among sampling points and depths (SF-surface, 20, 50, and 80 cm) by means of LDChip200 analyses (further information in Sánchez-García et al., 2018). Red color indicates positive antibody reaction (positive fluorescence signal), while white color indicates lack of positive reactions (i.e. presence-absence of positive immunoreaction).

All samples were analyzed for the presence of molecular content capable of interacting with a panel of antibodies in LDChip by a sandwich-type fluorescent microarray immunoassay (see section “Materials and Methods”). Because most LDChip antibodies were produced to bind to microbial strains from a variety of phylogenetic groups, the positive immunodetection signals were classified as a function of the microbial phylogeny of the strain used as immunogen. A heatmap showing the phylogenetic adscription of the positive immunodetections (Figure 6A) confirmed the trends elucidated above by the DNA analyses, with a broad distribution of bacterial and archaeal phyla and classes. LDchip detected markers from proteobacterial classes (*Alphaproteobacteria*, *Betaproteobacteria*, and *Gammaproteobacteria*), as well as *Actinobacteria* and *Firmicutes*, whose signals were higher in D8 samples. *Nitrospirae*-related markers were detected in higher abundance at D3, D6, and D10 samples, along with a remarkably strong signal for *Bacteroidetes* in these same locations. Halophilic archaeal cells showed a high signal in D8 samples, while archaeal methanogens were only detected in the D6-SF sample.

LDChip also detected proteins associated with certain metabolic processes (Figure 6B). Signals to stress response proteins (chaperones), iron storage (DPS protein also related to starvation), and nitrogen metabolism (mainly fixation) were detected at D3-20, while D8-80 showed a positive signal for a potential perchlorate reductase. A BLAST search of protein sequences detected by LDChip against data from the metaproteomic analysis identified chaperone GroEL and the structural protein FtsZ, two universal bacterial proteins, as found with both technologies.

## Discussion

### Soil Moisture Influences Geochemical Depth Profile

Parameters such as the pH of soil-water suspensions, nitrate and sulfate concentrations, and gravimetric moisture content showed certain trends with depth (down to 80 cm), a pattern of variation that has been recorded for similar parameters in nearby Atacama playas (~50 km due East) under drier conditions (Warren-Rhodes et al., 2019). Although the highest water content values were found at 10 or 20 cm depths, the values measured in deeper samples suggested that a small amount had also percolated below, as this moisture was always higher than compared with similar depths in other desert playas (Hang et al., 2015; McKenna and Sala, 2018) or desert soils (Neilson et al., 2017; Jones et al., 2018). In almost all sites, nitrate, sulfate, and formate concentrations increased with depth (Figure 2). It has been described that nitrate accumulated in hyperarid desert soils with reduced microbial growth (Díaz et al., 2016). Our results suggest that in the upper, more humid layers, denitrification processes could account for nitrate depletion, in agreement with Crits-Christoph et al. (2013) findings. In fact, metaproteomic analysis revealed the presence of a wide range of proteins for a potential nitrate metabolic assimilation, with an increased presence in these samples (see section “Results”). With depth and lower water content, nitrate accumulates, probably as a consequence of minor metabolic activity (Orlando et al., 2010) and/or the percolation effect. Additionally, a contribution to nitrate accumulation could also be made by ammonia oxidizing archaea (AOA) in all samples, especially at deeper layers.

**Figure 7:**
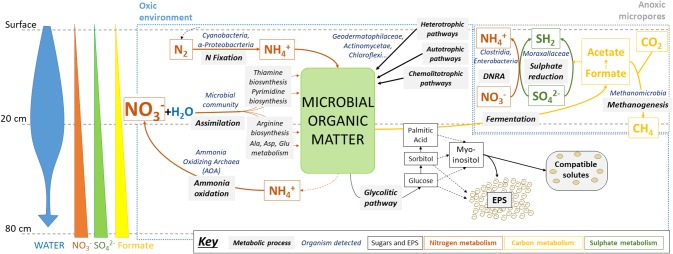
Microbial metabolisms in swallow subsurface of Atacama Desert playa environment after rainfall. Metabolic pathways were inferred from the results obtained after multi-analytical approach: ion chromatography (IC), sugar detection, high-throughput DNA sequencing, metaproteomics, and immunological detection with LDChip antibody microarray. Water input seems to be the main driver of microbial activity in oxic environments, mainly for N-cycling (orange arrows) and glycolytic pathways (dark thin arrows), as well as in creating anoxic environments, where S- (dark green arrows) and C-cycles (yellow arrows) could be operating, including CH_4_ production and consumption. See text for further explanation. Microbial groups referred as putatively responsible for metabolic pathways are based on both *16S* rRNA sequencing findings and previous studies that determined their metabolic capabilities (see section “Discussion”).

Lower formate concentrations in upper layers could be explained by its use as an electron donor for sulfate reduction and methanogenesis in anoxic micropores, for example by the detected *Methanobacteria* archaea, relatively abundant in these samples (see below) and potentially capable of carrying out these metabolic pathways (Gilmore et al., 2017). In turn, in these putatively metabolically-active niches, reduced sulfur compounds can be oxidized again by coupling to nitrate reduction, thus explaining their low accumulation (Figure 7).

### Microbial Community Structure Was Conditioned by the Transient Wet Habitat

Principal microbial groups identified in the study by both DNA sequencing and/or LDChip analyses resemble those described in earlier Atacama soil studies, with *Actinobacteria* and *Proteobacteria* being the most abundant phyla in upper soil layers and deeper ones respectively, while archaea were much less abundant than bacteria (Neilson et al., 2017; Schulze-Makuch et al., 2018; Warren-Rhodes et al., 2019). A decreasing trend in average bacterial diversity was observed with depth, similarly to what has been found in similar studies under dry conditions (Warren-Rhodes et al., 2019). Potential salt-forming ions (e.g. chloride and sulfate) are importantly present at these deeper samples (Supporting Information S5, S7), influencing microbial communities at these same depths (Figure 4A). Therefore, an inverse relation between bacterial diversity and salinity could be proposed, with only halotolerant bacteria favored by the environmental conditions at depth.

*Actinobacteria* phylum dominated the upper layers in all sites, similarly to other similar studies (Warren-Rhodes et al., 2019), with different families being most abundant in the playa (*Geodermatophilaceae*) versus the alluvial fan sites (*Actinomycetaceae* and *Acidomicrobiaceae*). Proteobacterial taxa were similar in D3 and D6 upper layers (wetter habitats), but differences were found for the deepest playa samples (80 cm) compared with all alluvial fan samples (dryer habitats). In fact, this latter alluvial fan site showed a similar microbial composition to nearby dry alluvial soils (Warren-Rhodes et al., 2019). However, the relatively high abundance of *Alphaproteobacteria* families *Erythrobacteriaceae* and *Sphingomonadaceae*, and the *Betaproteobacteria Comamonadaceae* and *Burkholderiaceae*, contrasts with previous reports considering these groups as “rare taxa” in arid soil environments (Aanderud et al., 2015; Vikram et al., 2016; Warren-Rhodes et al., 2019). Our results could be explained by what has been shown previously for the relative abundance of these families and their notable increase after the hydration of soils, including desert ones (Aanderud et al., 2015; Aslam et al., 2016). Therefore, even for common microbial phyla in desert soils, water input has a principal role in structuring different microbial communities.

Non-photosynthetic *Chloroflexi* were mainly present in the wet 20 cm depth playa samples, with OTUs identified as green non-sulfur, heterotrophic bacteria, in agreement to other sheltered habitats, such as hypolithic environments (Lacap et al., 2011) but again in contradiction to previously described communities from dry playa subsurfaces in the Atacama (Warren-Rhodes et al., 2019). *Moraxellaceae* family (*Pseudomonadales* order, *Gammaproteobacteria*) was clearly associated with the alluvial fan (D8) at that depth, as previously reported in other dry, gypsum-rich soils (López-Lozano et al., 2012). The higher gypsum (e.g. CaSO_4_·2H_2_O) concentrations measured at 20 cm depth from the alluvial fan in our study (D8) could favor the development of chemolithoautotrophic microorganisms involved in the sulfur cycle instead of the heterotrophic members of *Actinobacteria* and *Chloroflexi* that were observed in the playas. Although to a lesser extent in comparison to water influence, different geochemical parameters, such as the presence of different anions like sulfate, appear to be influencing bacterial group abundance in our samples.

The detection of obligated methanogenic archaea both with DNA (*Methanobacteria*, *Methanomicrobia*) and the LDChip, principally from just beneath the surface to 20 cm, suggests that methanogenic activity may occur in anoxic micropores created by water inputs, as has been described before (Angel et al., 2011). Further, the widespread presence of potential ammonia-oxidizing archaea (AOA) may indicate that relevant nitrification processes could be taking place in these newly created microaerobic microniches (see above). Therefore, although being less abundant than bacteria, archaeal communities in these environments seem to play a key role in ecosystem functioning. Again, water availability is the main factor driving these measured differences in microbial community composition and in putative metabolic activities in the analyzed samples. These detected microbial community variations in comparison to the one in dry areas that may resemble a pre-rain state (e.g. Warren-Rhodes et al., 2019) reinforce the main idea of rainfall is the major factor influencing the development of less desiccation tolerant taxa. The biomass content of these desert soils may also have increased from a pre-rain situation, especially at deepest soils. For instance, Warren-Rhodes et al. (2019) extracted much lower concentrations of environmental DNA in dry playa environments, also yielding a much minor microbial diversity. However, the lack of real information from our same soils in a pre-rain state persuades us not to make any further asseveration on microbial community changes.

Additionally, the diverse community structure at different environmental settings (i.e. playa versus alluvial fan) may have relevant implications for further microbial successions after sporadic wet events (Aanderud et al., 2015). Since samples were obtained in a very precise moment of ecosystem evolution, follow-up studies on similar desert environments would indicate whether the microbial communities evolved from an autotrophic-“seed bank” in the alluvial fan or from a heterotrophic one in playa soils, based on the ideas proposed by Fierer et al. (2010) for community development. Alternatively, such posterior studies could show the actual situation in both sites was just a transitory habitat created by the extra water supply, as has been proposed before (Schulze-Makuch et al., 2018) or a part of a microbial community two-step adaptation to an environmental change, as it has been found for halite-rich Atacama soils (Uritskiy et al., 2018).

### Proteins for Primary Metabolic Functions Were Present in All Samples

Although scarce, proteins were detected in all samples, with a minimum of 0.31 ppm in D1-80 and a maximum of 130 ppm in D8-50, with no obvious trend with depth or sampling site. The identification of proteins in desert soils is a reliable method of inferring metabolic capabilities of the microbial community. In the absence of macrobiota in desert habitats, the role of microbial metabolisms is even more relevant to the maintenance of biogeochemical cycles (Bastida et al., 2014; Makhalanyane et al., 2015). Proteins are target biomarkers in surface and subsurface dry environments because of their relative stability, enhanced by xeropreservation processes (Parro et al., 2011). Proteins and peptides also interact with minerals (Schulten, 1995), which further enhances their preservation potential over time. These factors are some of the multiple reasons the Atacama Desert is considered a model for testing methods and instruments for extinct life detection on Mars (Parro et al., 2011). Following that, metaproteomic results also allow the study of both recently produced and well-preserved proteins, with the most abundantly detected proteins expected to be those involved in the most active and common metabolisms (energetic metabolisms, biosynthesis, replication, protein and nucleic acid folding, etc.).

Results from our metaproteomic and LDChip study analyses revealed a dominance of primary metabolism, with basic biosynthetic and metabolic activities (from chemolitoautotrophy to organoheterotrophy) related to major biogeochemical cycles, particularly the nitrogen cycle. On the one hand, the detected widespread distribution of proteins detected for nitrate and nitrogen assimilation, as well as proteins for nitrogenized compounds biosynthesis coupled to variations in nitrate content along different depths, could point to active nitrogen cycling by the microbial community, likely enhanced by water availability, as proposed previously (Díaz et al., 2016; Neilson et al., 2017). Alternatively, the detection of specific (notably at SF and 20 cm samples) proteins involved in nitrogen fixation, such as NifH and NifD in the upper soil layers (SF and 20 cm), may also highlight the key ecological role of nitrogen fixers, which provide nitrogenous compounds to the system (Makhalanyane et al., 2015). In these samples, most of the nitrate could have already been incorporated by non-fixing microorganisms shortly after the water input, thus favoring nitrogen fixation as a separate pathway for N-cycling.

Enzymes involved in sulfur metabolisms, such as for sulfate reduction to H_2_S (e.g. sulfate adenylyltransferase), were mainly detected in shallower samples, where water could have created anoxic micropores and a reductive metabolism could be coupled to a dissimilatory nitrate reduction, as discussed above. Small organic acids as acetate and formate from anaerobic fermentation of organic matter in these anoxic microenvironments could, in turn, be used as a substrate for sulfate reduction.

Another principal contribution to organic matter content was the exopolysaccharidic (EPS) fraction. We detected glucose, galactose, sorbitol and palmitic acid, which could be directly related to recent or active glycolytic pathways, as some of the enzymes involved have been detected. As a first step in glucose metabolism, this sugar is reduced to sorbitol, which can be converted to fructose (an intermediate compound in the glycolytic pathway) or to galactose. Further, all these sugars can derive into the key central metabolism compound acetyl-CoA which finally could be converted into fatty acids such as palmitic acid, the first compound to be formed in most lipid biosynthetic pathways *via* glycolysis. We also detected myoinositol in the EPS fraction, which is in agreement with the detection of an enzyme specifically involved in its biosynthesis (inositol-phosphate phosphatase) in the metaproteome. Myoinositol can be part of the intracellular pool of compatible solutes (Empadinhas and Da Costa, 2009) or as the extracellular EPS fraction as a mechanism to deal with high salt or desiccation conditions (Martínez-Cánovas et al., 2004).

Altogether, our data suggest a metabolically active or very recently active microbial community after the rainfall, in agreement with more favorable post-rainfall habitat conditions reported for other sites in the Atacama (Schulze-Makuch et al., 2018). As far as we know, a metaproteomic profile similar to our study has only been described for less harsh, semiarid soil environments (Bastida et al., 2014) or in active-suggested desert-like microbial communities (Dorado-Morales et al., 2016). The low number of stress-related proteins quantified in our study (less than 2% of total proteins – including those identified by a distinct KEGG – and a restricted number of positive immunosignals in LDChip – including those also identified in the metagenomic study by means of the BLAST analysis) contradicted the relatively high abundance of proteins involved in stress response or dormancy previously postulated for desert arid environments (Fierer et al., 2012). We hypothesize that only those habitats with long transitions from wet to dry conditions, with microorganisms subjected to gradually increasing stressful environments, would show a considerable metaproteomic content toward specific pathways and stress resistance mechanisms. Again, the lack of information on the pre-rain state of these same soils prevent us to make any further asseveration on the capability of pre-existing microbial communities and their metabolic activity. However, subsequent studies taking into account the environmental evolution from the moment of sampling of this study would shed light on our hypothesis. Therefore, following our results we can suggest that (1) the greater water availability within days of the rainfall event provided the microbial community with temporarily milder conditions characterized by adequate available nutrients, less radiation (mainly in the deepest layers), mild temperatures, and minimal oxidative and hydric stresses; and (2) the microbial community responded rapidly with active growth metabolisms at all depths and habitats.

Following Fierer et al. (2007), the two main growth strategies in community ecological theory that can be directly applied to microorganisms are the “r-strategy” – devoted to increasing the population, typical of microorganisms better adapted to changing environments, i.e. “microbial generalists” – or the “K-strategy” – devoted to competing for limited resources, that is, strategies more typical of “microbial specialists”, which tend to be more vulnerable to environmental change (Uritskiy et al., 2018). The finding of a high abundance of microbial groups characterized as “generalists” in these soils, such as families within *Proteobacteria* or *Actinobacteria*, as well as the remarkable increase of betaproteobacterial infrequent groups, in comparison to the lower abundance of microbial specialists (restricted to specific samples in the study, see section “Results” and previous subsection in “Discussion”) may demonstrate that during the transitory wet habitat, the Atacama soil microbial communities followed an “r-strategy.” This key ecological finding is further supported by the detection of a high abundance of proteins involved in antibiotic production, which has been previously reported to be intra-specific signaling to induce efficient developmental pathways rather than signaling inter-specific competition (Bull et al., 2016; Mohammadipanah and Wink, 2016; Noronha et al., 2017). In fact, these signaling proteins seem to be more abundant in nutrient-rich environments (Hibbing et al., 2010; Fierer et al., 2012), in agreement with mild, non-stressful conditions. However, a progressive decline over time in microbial development is expected if succession toward a hyperarid environment occurs, thus leading to a nutrient scarcity and a microbial populations’ balanced coexistence (Ghoul and Mitri, 2016). Consequently, our research sheds light on ecological succession in desert soil microbial communities during a water-induced population growth and an eventual return to stressful conditions and growth-limited survival strategy.

## Concluding Remarks

The combination of geochemical, DNA sequencing, and metaproteomic analyses of environmental samples in this study allowed inferences about the structure and nature of the microbial populations, their diversity, and their putatively metabolic states to be elucidated for small Atacama playas and an alluvial fan after a sporadic but large-scale rain event. The data revealed a metabolic scenario dominated by primary metabolic processes such as energetic and biosynthetic pathways (especially nitrogen metabolism) and other functions involved in ecological interactions mediated by antibiotic production. Although minor, stress-related functions were also identified though LDChip and metaproteomics. Further research on the secondary succession and adaptation of microbial communities following a large precipitation event, as well as biomarker preservation in hyperarid Atacama soils, is required. Our data may also furnish insights into how a heretofore transitory microbial response to a rainfall event might shift with a progressive increase in regional water availability as a consequence of climate change.

The metaproteomic analysis also enabled an estimate of the concentration of proteins in the soil to certain depths. The presence of detectable proteins and a set of trypsin-digested peptides infer relatively well-preserved biological material. This sampling time-point and its results can thus be considered a starting point to follow community structure and protein stability and its preservation potential. Considering the Atacama Desert is one of the best analogues for addressing the feasibility and strategies for the search for life on Mars, our biogeochemical results depict an analogue scenario for studying the biomolecular record and its preservation in the context of an evolving “early wet” Mars, where sporadic wet events were followed by long hyperarid periods (Fairén et al., 2010). LDChip detection and profiling of microbial markers, even at the level of protein and peptides, provided a wealth of information in the current study, reinforcing the potential for its successful application in future life detection missions to Mars.

## Data Availability

The datasets generated for this study can be found in NCBI Sequence Read Archive, PRJNA506644.

## Author Contributions

MF-M carried out data analyses and wrote the manuscript. RS carried out DNA and protein extraction. IG-C collected soil samples, prepared samples for ion chromatography and carried out LDChip200 immunoassays. YB and MM-P carried out LDChip200 immunoassays and analyses as well as contributed to improve the manuscript edition. MV performed ion chromatography analyses and DNA and protein extraction. AB advised on statistical analyses as well as discussed phylogenetic results and contributed to the manuscript edition. MR-B performed sugars, alcohols, and fatty acids identification by means of GC-MS analyses. KW-R, DW, NC and VP designed the study, the sampling strategy, participated in fieldwork and contributed to improve the manuscript edition.

### Conflict of Interest Statement

The authors declare that the research was conducted in the absence of any commercial or financial relationships that could be construed as a potential conflict of interest.

## Footnotes

^1^www.matrixscience.com
