# Supplementary material for: Prokaryotic Community Structure and Metabolisms in Shallow Subsurface of Atacama Desert Playas and Alluvial Fans After Heavy Rains: Repairing and Preparing for Next Dry Period
Source: Front Microbiol. 2019 Jul 24;10:1641. doi: 10.3389/fmicb.2019.01641 (PMC6668633; doi:10.3389/fmicb.2019.01641)
Supplement: Supplementary file 1 [file Data_Sheet_1.PDF]

SUPPORTING INFORMATION

Prokaryotic community structure and metabolisms in shallow subsurface of Atacama Desert playas and alluvial fans after heavy rains: repairing and preparing for next dry period.

Miguel Ángel Fernández-Martínez(1)*, Rita dos Santos Severino(1), Mercedes Moreno Paz(1), Ignacio Gallardo Carreño(1), Yolanda Blanco(1), Kimberley Warren-Rhodes(2), Miriam García Villadangos(1), Marta Ruiz-Bermejo(1), Albert Barberán(3), David Wettergreen(4), Nathalie Cabrol(2) and Víctor Parro(1).

[1] Centro de Astrobiología (CAB, CSIC-INTA), Madrid, Spain

[2] SETI Institute, Carl Sagan Center, Mountain View, California, USA; NASA Ames Research Center, Moffett Field, California, USA

[3] Department of Soil, Water, and Environmental Science, University of Arizona, Tucson, AZ, USA

[4] Carnegie Mellon University, Robotics Institute, Pittsburgh, PA 15213, United States

* Corresponding author, email: mafernandez@cab.inta-csic.es, telephone: +34 520 16 33

Supporting information S1. Sugars, alcohols and fatty acids identification

Following steps were carried out for the identification:

(i) The extracted samples for polysaccharides were dialyzed intensively against ddH_2O, freeze-dried and a weight corresponding to 100 µg of total carbohydrates was hydrolyzed with 6 M HCl at 75 ºC overnight and again freeze-dried to remove water and HCl.

(ii) Hydrolyzed samples in 75 µL of Power Syl-Prep [TMSIM (N-Trimethylsilylimidazole): BSA: TMCS (Trimethylchlorosilane) (3:3:2), from Alltech (Grace, Deerfield, IL, USA)] were heated at 70 ºC for 15 min to obtain the corresponding TMS derivatives.

(iii) Derivatized samples were extracted with 400 µL of water and 100 µL of hexane.

(iv) Organic layers were collected and analysed by GC-MS using 2 µL for each sample and following the GC oven program specifications:

   a. 100 ºC (initial temperature) with a hold of 1 min.
   b. Heat to 200 ºC at 30 ºC min^{-1} with a hold time of 15 min.
   c. Heat to 270 ºC at 15 ºC min^{-1} with a hold time of 20.

(v) The temperature of the injector was 300 ºC, and the injections were made in splitless mode. The detector temperature was 300 ºC, and the flow rate was 1.1 mL min^{-1}. The GC-MS analyses were done in the full-scan mode and were carried out on a 6850 network GC system coupled to a 5975 VL MSD with a triple-axis detector operating in electronic impact mode at 70 eV (Agilent Technologies), with an HP-5MS column (30 m x 0.25 mm i.d. x 0.25 mm film thickness) and He as carrier gas.

As a rule, the identification of monosaccharides, related compounds and fatty acids by the GC-MS peaks was verified by comparison with the retention times and mass spectra of external standards, purchased from Sigma-Aldrich. In the case of no available standards the identification of the analytes were made using the NIST 2014 MS Library (Agilent Tehcnologies) with a match above 90%.

Supporting information S2. PCRs and Illumina sequencing specifications.

Purified DNAs were quantified by Picogreen and 200 pg of input DNA were used in a first PCR with Q5® Hot Start High-Fidelity DNA Polymerase (New England Biolabs) (26 cycles) in the presence of 100nM primers for bacterial *16S* rDNA gene region amplification (341-F / 805-R primer pair) or in the presence of 100nM primers for archaeal *16S* rDNA gene region amplification (Arch1F / Arch1R primer pair).

After the first PCR, a second PCR was perfomed with Q5® Hot Start High-Fidelity DNA Polymerase (New England Biolabs) (15 cycles) in the presence of 400nM of primers (5'–AATGATACGGCGACCACCGAGATCT-ACACTGACGACATGGTTCTACA-3' and 5'-CAAGCAGAAGACGGCATACGAGAT-[barcode]–TACGGTAGCAGAGACTTGGTCT-3') of the Access Array Barcode Library for Illumina Sequencers (Fluidigm).

The obtained amplicons were validated and quantified by an Agilent 2100 Bioanalyzer using DNA7500 chips. Then, an equimolecular pool of these amplicones was purified with AMPure-XP Beads (Beckman coulter) to eliminate primers/dimers and titrated by quantitative PCR using the "Kapa-SYBR FAST qPCR kit forLightCycler480" and a reference standard for quantification. The pool of amplicons were denatured prior to be seeded on a flowcell at a density of 10pM, where clusters were formed and sequenced using a "MiSeq Reagent Kit v3", in a 2x300 pair-end sequencing run on a MiSeq sequencer.

Supporting information S3. Detailed Metaproteomics analyses.

After protein extraction with GuHCl buffer, extracts were dialyzed (>1200 Da cutoff) against distilled water and finally lyophilized. These extracts were then used for total protein quantification using Quant-iT™ Protein Assay kit (Invitrogen, Oregon, USA) according to manufacturer's instructions as well as read on Qubit™ Fluorometer (Invitrogen, Oregon, USA). The Total sugar content in these same extracts was determined by means of the phenol-sulphuric method (Dubois et al., 1956).

These protein extracts were dissolved in 200 µL of 8 M Urea/2M thiourea under heat. 20 µL of SDS-PAGE loading buffer was later added to 100 µL of protein solution and the mixture was loaded into a 4% SDS-PAGE stacking gel, 10% separation. Electrophoresis stopped when the front-run was at 3 mm before entering the separation gel. Gel was then stained with colloidal coomasie prior to cut out protein bands, that subsequently were subjected to direct trypsin digestion (by incubation with 10 mM DTT at 56ºC for 30 min to reduce them, then at 55mM Iodacetamide for 20 min in the dark for their alkylation, and finally digested with 1.5 µg of Trypsin, sequencing grade - Roche Molecular Biochemicals - in 25 mM ammonium bicarbonate pH 8.5 for 12 h at 37ºC). Obtained tryptic peptides were desalted and concentrated by C18 reverse chromatography in the tip (OMIX, Agilent technologies), lyophilized and frozen until their analysis using LC-MS. Processed peptides were then analyzed by nano-liquid chromatography (Eksigent Technologies nanoLC Ultra 1D plus, ABSCIEX, Foxter City, CA) coupled to a high resolution mass spectrometer 5600 triple TOF (ABSCIEX) in the Centro de Genómica y Proteómica, Universidad Complutense de Madrid (Madrid, Spain).

The following parameters were used for searching protein identities with MASCOT software against Swissprot database: tryptic cleavage after Arg and Lys, up to two missed cleavage sites allowed, and tolerances of 20 ppm for precursor ions and 0.8 Da for MS/MS fragment ions. The searches were performed allowing optional Methionine oxidation and fixed carbamidomethylation of Cysteine. Search against decoy database (integrated decoy approach) was used to FDR calculate MASCOT percolator filter were applied to MASCOT results. The acceptances criteria for proteins identification were a FDR < 1% and at least one peptides identified with high confidence (CI>99%).

Supporting information S4. Mineralogical composition (mineral presence or absence) of soils obtained by powder X-Ray diffraction analyses (XRD).

Sample	Phyllosilicates and sillicates													S	P	Iron		U	Mn
	Quartz	Albite	Olivine	Mica	Microcline	Muscovite	Anorthite	Annite	Takanelite	Pargasite	Clinochlore	Zinnwaldite	Katayamalite	Gypsum	Scorzalite	Hematite	Orthopyroxene	Billietite	Kutnahorite
**D1-SF**	✓	✓																	
**D1-10**	✓	✓																	
**D1-20**	✓	✓																	
**D1-50**	✓	✓											✓			✓			
**D1-80**	✓	✓												✓					
**D3-SF**	✓	✓																	
**D3-10**	✓					✓	✓												
**D3-20**	✓	✓						✓											
**D3-50**	✓	✓			✓														
**D3-80**	✓	✓							✓										
**D6-SF**	✓	✓		✓															
**D6-10**	✓	✓																	
**D6-20**	✓	✓																	
**D6-80**	✓	✓																	
**D8-SF**	✓	✓			✓														
**D8-10**	✓	✓			✓														
**D8-20**	✓	✓												✓					
**D8-50**	✓													✓	✓		✓		
**D8-80**	✓	✓			✓									✓					
**D10-SF**	✓	✓								✓						✓			
**D10-10**	✓	✓								✓	✓	✓							
**D10-20**	✓	✓										✓							
**D10-50**	✓	✓																	
**D10-80**	✓	✓																✓	
**D16-SF**	✓					✓	✓												
**D16-10**	✓						✓												
**D16-20**	✓	✓																	✓
**D16-50**	✓	✓	✓																
**D16-80**	✓	✓			✓														

Formulas: Quartz: SiO_2; Albite: $Na(AlSi_3O_8)$; Olivine: $(Mg, Fe)_2SiO_4$; Mica: $AB_{2-3}(X, Si)_4O_{10}(O, F, OH)_2$; Microcline: $K(AlSi_3O_8)$; Muscovite: $KAl_2(AlSi_3O_{10})(OH)_2$; Anorthite: $Ca(Al_2Si_2O_8)$; Annite: $KFe^{2+}{}_3(AlSi_3O_{10})(OH)_2$; Takanelite: $(Mn, Ca)Mn_4O_9 \cdot H_2O$; Pargasite: $(Na, Ca_2, Mg_4Al)(Al_2Si_6O_{22})(OH)_2$; Clinochlore: $Mg_5Al(AlSi_3O_{10})(OH)_8$; Zinnwaldite: $KLiFe^{2+}Al(AlSi_3O_{10})(F,OH)_2$; Katayamalite: $KLi_3Ca_7Ti_2(SiO_3)_{12}(OH)_2$; Gypsum: $CaSO_4 \cdot 2H_2O$; Scorzalite: $Fe^{2+}Al_2(PO_4)_2(OH)_2$; Hematite: Fe_2O_3; Orthopyroxene: $(Mg, Fe, Mn)Si_2O_6$; Billietite: $Ba(UO_2)_6O_4(OH)_6 \cdot 4\text{-}8H_2O$; Kutnahorite: $Ca(Mn, Mg, Fe)(CO_3)_2$; Bracewellite: $CrO(OH)$.

Supporting information S5. Physicochemical composition of soils obtained by ion chromatography (IC)

Sample	TotalProtein (μg/g)	TotalSugar (μg/g)	Fluoride (ppm)	Acetate (ppm)	Formate (ppm)	Chloride (ppm)	Nitrate (ppm)	Nitrite (ppm)	Sulphate (ppm)	Bromide (ppm)	pH	% water
**D1-SF**	3.86±0.030	1.5±0.021	8.74±0.075	2.02±0.021	0.58±0.061	3.02±0.001	26.46±1.257	0	9.24±0.129	0	7.51±0.651	0.586±0.061
**D1-10**	0.4±0.0120	4.06±0.022	18.81±0.421	28.75±0.176	0	3.96±0.015	0	0	804.76±29.721	0	8.02±0.721	4.154±0.391
**D1-20**	0.38±0.004	5.13±0.056	15.32±0.854	0.94±0.008	0	13.68±0.124	168.52±3.566	0	1021.86±21.336	0	7.92±0.771	4.447±0.522
**D1-50**	0.45±0.002	4.31±0.026	23.45±0.406	7.58±0.155	3.44±0.134	7.56±0.231	85.94±2.927	0	1597.89±52.388	0	7.85±0.677	0.993±0.089
**D1-80**	0.31±0.002	4.33±0.031	26.6±0.613	13.57±0.252	3.61±0.146	8.28±0.352	61.32±2.018	0	24652.12±772.531	0	7.72±0.653	0.35±0.032
**D3-SF**	17.5±1.236	38.89±3.401	17.45±0.363	5.78±0.012	0.78±0.008	1.91±0.057	5.85±0.188	0	11.45±0.436	0	7.3±0.713	0.584±0.064
**D3-10**	39.75±1.560	32.66±2.712	20.07±0.602	3.86±0.117	1.54±0.019	23.23±0.771	222.85±8.244	0	3554.46±120.191	13±0.015	7.59±0.751	1.652±0.171
**D3-20**	34.98±1.643	19.55±1.751	29.08±0.752	6.66±0.058	2.7±0.034	46.12±1.463	882.18±5.367	0	429.08±3.217	0	7.99±0.811	0.254±0.024
**D3-50**	17.75±0.984	16.49±1.543	28.75±0.602	9.05±0.165	3.42±0.024	17.08±0.554	171.35±5.652	0	7127.42±226.459	0	7.79±0.698	0.213±0.022
**D3-80**	52.27±2.012	10.66±1.006	32.55±0.824	10.45±0.117	2.98±0.030	22.47±0.694	233.76±7.787	0	10320.71±327.801	0	7.89±0.802	0.386±0.041
**D6-SF**	30.79±1.520	6.96±0.548	15.95±0.257	5.28±0.062	0.52±0.005	5.89±0.098	19.26±0.623	0	11.12±0.366	0	7.19±0.722	0.483±0.033
**D6-10**	4.76±0.273	17.03±1.683	19±0.311	10.6±0.012	0	21±0.174	117.8±1.334	0	1858.8±10.467	0	7.38±0.744	2.026±0.201
**D6-20**	18.31±1.021	10.77±1.523	36.2±0.568	6.2±0.071	2.6±0.003	103.4±0.354	1720.8±4.215	0	365.2±1.263	0	7.4±0.699	0.51±0.049
**D6-50**	4.27±0.321	13.23±1.278	14.2±0.0.186	6.8±0.056	2.4±0.002	562.8±3.336	4074.8±6.215	0	5841±17.368	0	7.63±0.759	0.263±0.024
**D6-80**	41.07±1.273	24.91±2.324	14.27±0.208	5.05±0.139	2.34±0.057	1125.07±37.521	10109.17±332.786	0	9904.01±317.369	0.96±0.003	7.28±0.712	0.383±0.036
**D8-SF**	29.29±1.026	5.61±0.482	11.1±0.069	6.73±0.087	1.23±0.017	4.61±0.007	4.93±0.045	0	6.78±0.192	0	6.77±0.675	0.167±0.022
**D8-10**	89.4±1.893	1.23±0.013	16.4±0.127	4.4±0.041	0	6.6±0.078	5.4±0.006	0	10±0.714	0	7.49±0.751	0.489±0.051
**D8-20**	48.3±1.231	6.3±0.571	4.58±0.041	1.72±0.016	0.86±0.007	3.47±0.019	7±0.004	0	28762.07±911.706	0	7.4±0.741	1.884±0.167
**D8-50**	130.33±2.254	18.05±1.651	23.91±0.493	5.39±0.194	0	16.01±0.541	180.34±7.844	0	30587.43±959.403	0	7.99±0.811	0.448±0.053
**D8-80**	68.48±2.032	8.7±0.793	28.2±0.535	3.6±0.042	3.01±0.002	32.6±1.591	274.6±6.223	0	30952.21±36.245	0	7.86±0.791	0.333±0.024
**D10-SF**	0.83±0.001	4.21±0.041	11.05±0.143	8.83±0.181	4.28±0.031	3.02±0.004	9.69±0.319	0	5.75±0.306	0	7.75±0.771	0.406±0.031
**D10-10**	22.28±1.364	6.58±0.711	27.54±0.248	3.36±0.044	0.52±0.006	3.1±0.012	22.98±2.265	0	17.02±0.153	0	8.68±0.859	1.938±0.175
**D10-20**	13±1.001	3.88±0.045	6.9±0.063	0.84±0.091	0.2±0.001	1.2±0.011	15.56±1.163	0	4.28±0.236	0	8.94±0.897	3.254±0.316

**D10-50**	2.17±0.021	1.88±0.021	0.56±0.006	0	0.44±0.003	4.78±0.051	1211.44±5.468	0	226.02±1.256	0.44±0.012	7.4±0.733	0.345±0.042
**D10-80**	35.95±1.041	11.02±0.126	3.16±0.0.35	1.12±0.002	1.04±0	76.86±2.003	10767.31±11.368	0	3059.94±12.472	0	6.93±0.685	0.907±0.102
**D16-SF**	3.11±0.030	3.73±0.322	16.08±0.245	0.96±0.025	0	0.54±0.002	4.94±0.006	0	17.58±0.186	0	7.81±0.779	0.2±0.019
**D16-10**	2.59±0.021	1.86±0.015	23.9±0.441	0.8±0.007	0.3±0.001	8.94±0.123	139.12±1.689	0	240.14±0.214	0.84±0.005	8.5±0.844	0.718±0.069
**D16-20**	6.79±1.023	2.82±0.033	29.64±0.386	1.08±0.012	0.22±0.002	30.58±3.143	393.98±2.684	0	920.06±1.965	0.82±0.006	8.01±0.799	0.145±0.015
**D16-50**	3.37±0.050	5.15±0.061	28.18±0.435	0.46±0.004	0.34±0.003	423.62±8.103	7074.28±15.459	0	1436.18±6.245	3.72±0.151	7.69±0.695	0.266±0.031
**D16-80**	7.08±0.958	3.41±0.033	15.76±0.179	0	0	176.6±1.214	3487.4±7.632	0.6±0.006	5805.92±12.356	0	7.74±0.688	0.172±0.023

Supporting information S6. Presence of monosaccharides and related compounds among samples and depths

	Monosaccharides			Polyalcohols			Fatty acids	
	**Galactose**	**Glucose**	**Manose**	**Sorbitol**	**Mannitol**	**Myo-Inositol**	**Palmitic acid**	**Stearic acid**
**D3-SF**		✓		✓			✓	
**D3-10**		✓		✓			✓	
**D3-20**	✓	✓	✓	✓	✓	✓	✓	
**D3-50**	✓	✓	✓	✓			✓	✓
**D3-80**	✓	✓	✓	✓		✓	✓	✓
**D6-SF**	✓	✓	✓	✓			✓	✓
**D6-10**	✓	✓	✓	✓		✓		
**D6-20**	✓	✓	✓	✓			✓	✓
**D6-50**	✓	✓	✓	✓				
**D6-80**	✓	✓	✓	✓				
**D8-SF**		✓						
**D8-10**		✓						
**D8-20**		✓					✓	✓
**D8-50**								
**D8-60**		✓					✓	

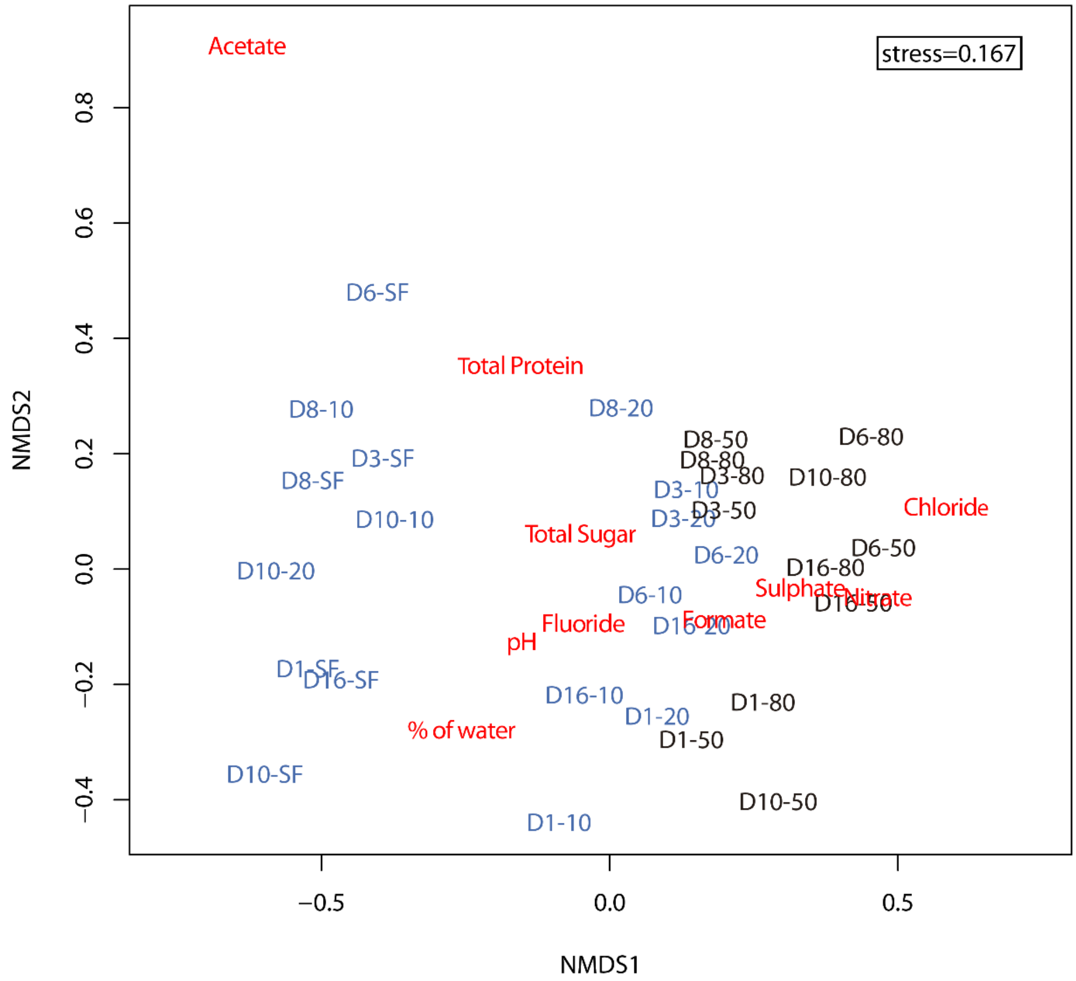

Supporting information S7. Two dimensional non-metric multidimensional scaling (NMDS) ordination plot of sampling points (based on Bray-Curtis similarities) showing the relationships among them and the analyzed physicochemical factors (in red) (stress = 0.164). Shallow samples (from surface - SF - and 10 cm depth) and intermediate depth samples (20 cm depth) are shown in light blue. Deeper samples (50 and 80 cm depth) are shown in black. Nitrite and Bromide were excluded from analyses due to their very scarce detection in the samples.

Supporting information S8. Soil microbial community composition as depicted by *16S* rRNA gene sequencing. Proteobacteria, Euryarchaeota and Thaumarchaeota phyla were divided into different classes to show further information.

	D3-SF		D3-20		D3-80		D6-SF		D6-20		D6-80		D8-SF		D8-20		D8-80	
	Seqs	OTUs	Seqs	OTUs	Seqs	OTUs	Seqs	OTUs	Seqs	OTUs	Seqs	OTUs	Seqs	OTUs	Seqs	OTUs	Seqs	OTUs
Actinobacteria	76513	0	73652	332	79252	666	71805	601	63055	347	2206	16	65733	317	29707	155	14603	262
Alphaproteobacteria	7315	142	64	3	5259	94	18113	159	1226	9	11633	44	5864	55	10660	56	51017	200
Betaproteobacteria	66	13	388	4	72	5	19	5	1015	11	28779	138	518	17	1210	14	25679	117
Gammaproteobacteria	345	12	893	5	294	14	66	8	644	6	3387	25	7	1	13491	94	70	3
Deltaproteobacteria	103	5	470	4	2	1	7	2	191	5	12	1	227	22	0	0	63	7
Epsilonproteobacteria	0	0	0	0	2	1	5	1	0	0	664	4	0	0	0	0	0	0
**Unclassified Proteobacteria**	2	1	0	0	0	0	0	0	0	0	15	2	108	10	2	1	12	1
Firmicutes	633	47	2791	33	195	26	156	27	1982	25	15283	71	2	1	30014	164	202	8
Chloroflexi	3768	103	3877	25	3969	94	2442	71	19250	63	2	1	5109	41	511	3	184	15
Bacteroidetes	397	22	24	3	198	9	97	11	1654	10	11562	50	3708	45	3313	25	184	12
Gemmatimonadetes	1697	29	6069	36	1553	23	540	18	629	9	0	0	6379	31	47	1	1762	28
Cyanobacteria	0	0	0	0	2	1	0	0	0	0	30	14	0	0	4	2	0	0
Acidobacteria	189	11	0	0	210	9	72	7	657	5	0	0	259	5	2147	18	475	15
Candidatus Saccharibacteria	0	0	0	0	0	0	0	0	134	2	14	1	902	12	820	7	97	11
**Others**	360	8	73	4	162	5	364	12	873	9	1092	4	205	9	774	13	219	19
**Unknown Bacteria**	4359	193	7416	47	4569	166	1986	116	4027	71	1641	39	6736	208	423	7	1140	103
Halobacteria	29	1	4	1	0	0	0	0	112	1	0	0	0	0	0	0	0	0
Methanobacteria	98	1	0	0	23	2	43	1	0	0	0	0	205	1	0	0	6	1
Methanomicrobia	30	3	0	0	0	0	2	1	105	1	0	0	2	1	6	3	6	2
Nitrososphaeria	12	6	6	3	8	8	2	1	4	2	4	2	10	5	225	12	12	3
**Unclassified Archaea**	0	0	0	0	0	0	2	1	0	0	0	0	0	0	54	1	0	0

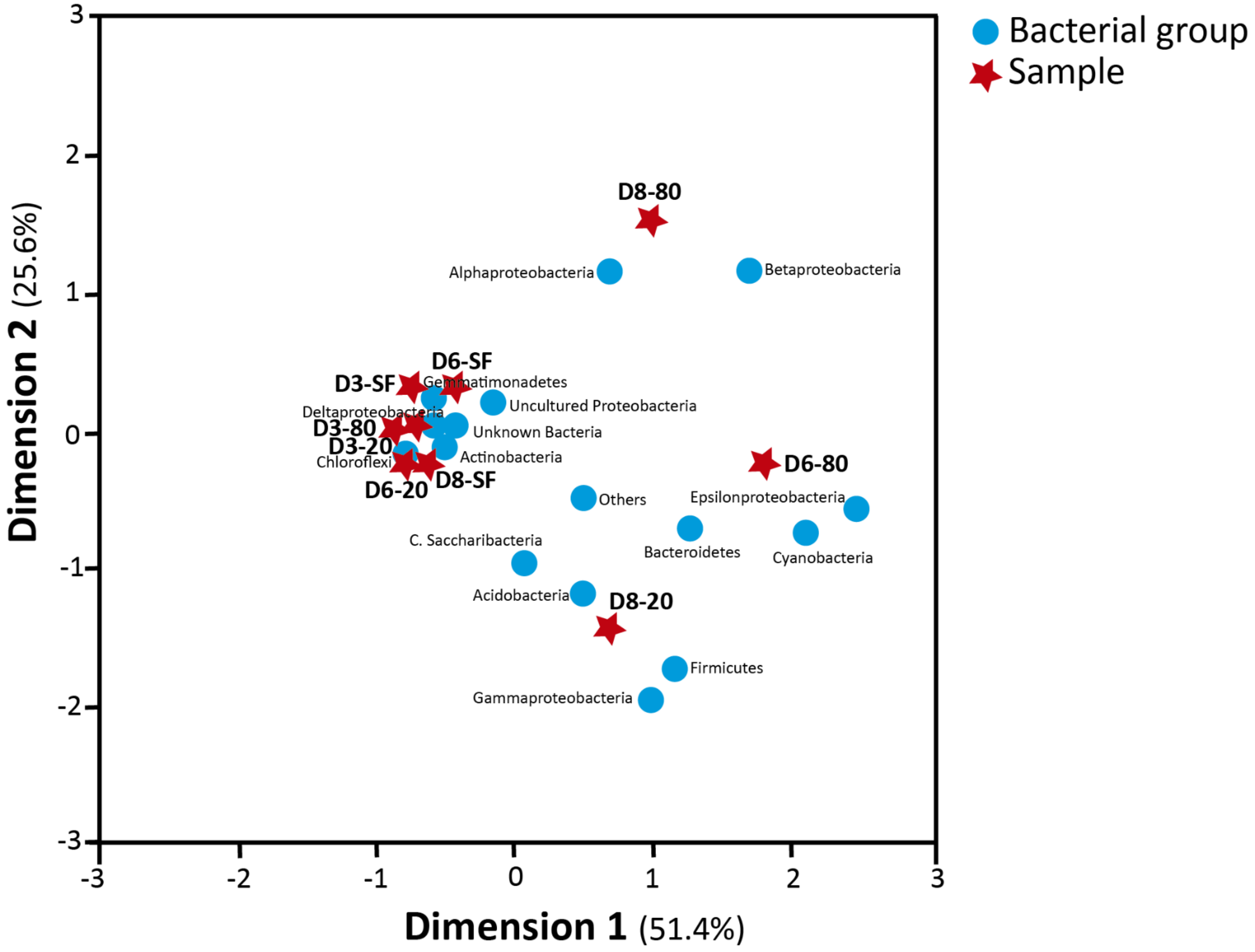

Supporting Information S9. Single correspondence analysis (CA) of bacterial classes (blue dots) according to samples (red stars). Variables explained 77% of the variance of the data set ($p \leq 0.001$).

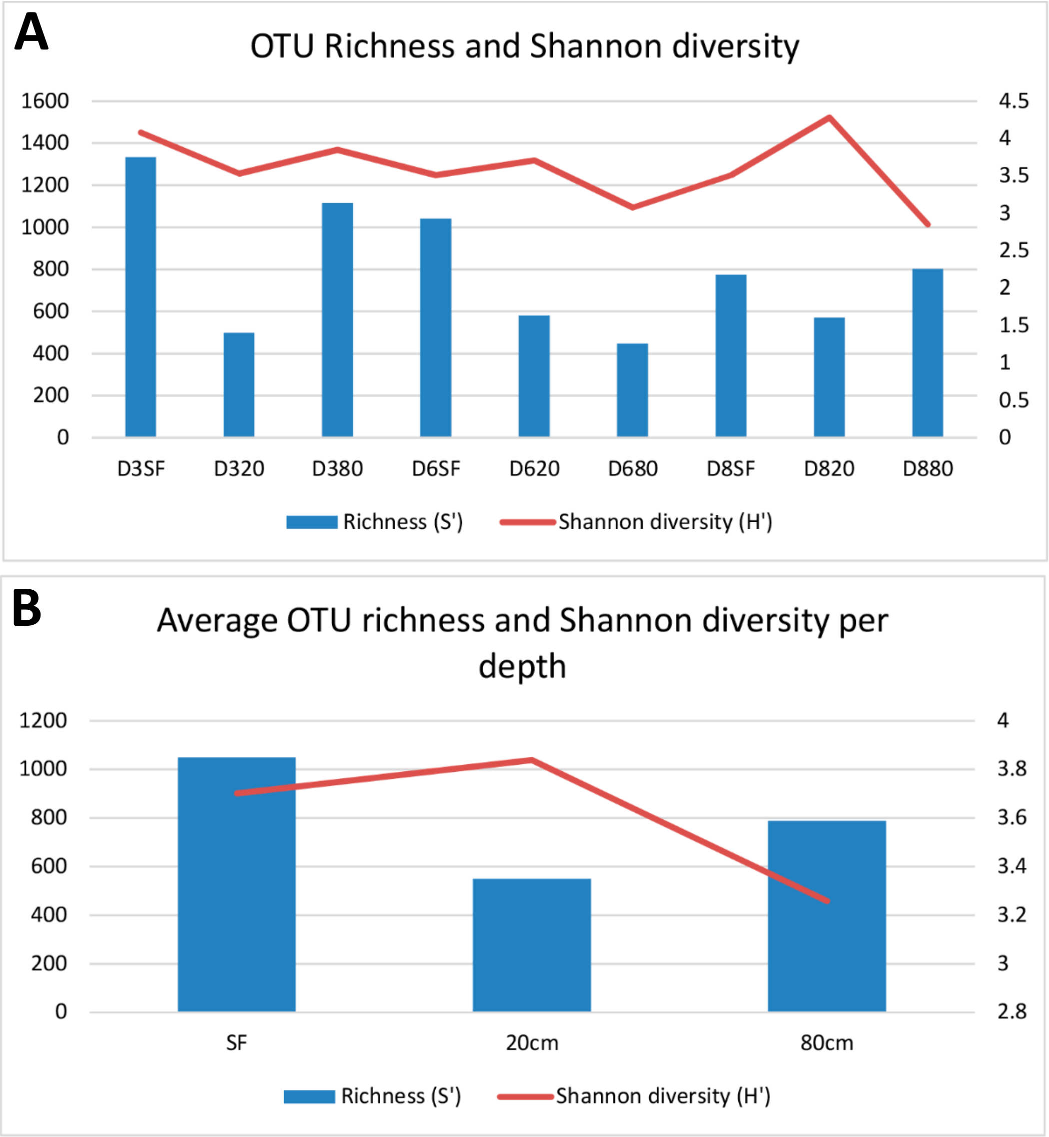

Supporting information S10. Bacterial OTU richness (*S'*) and Shannon diversity index (*H'*) (A) per sample and (B) on average per depth.

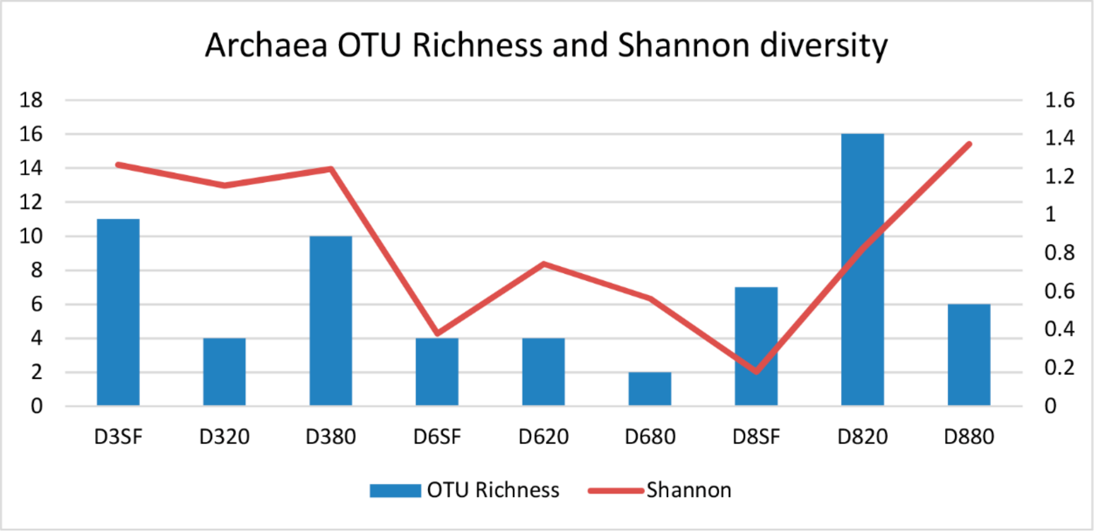

Supporting information S11. Archaeal OTU richness (*S'*) and Shannon diversity index (*H'*) per sample.

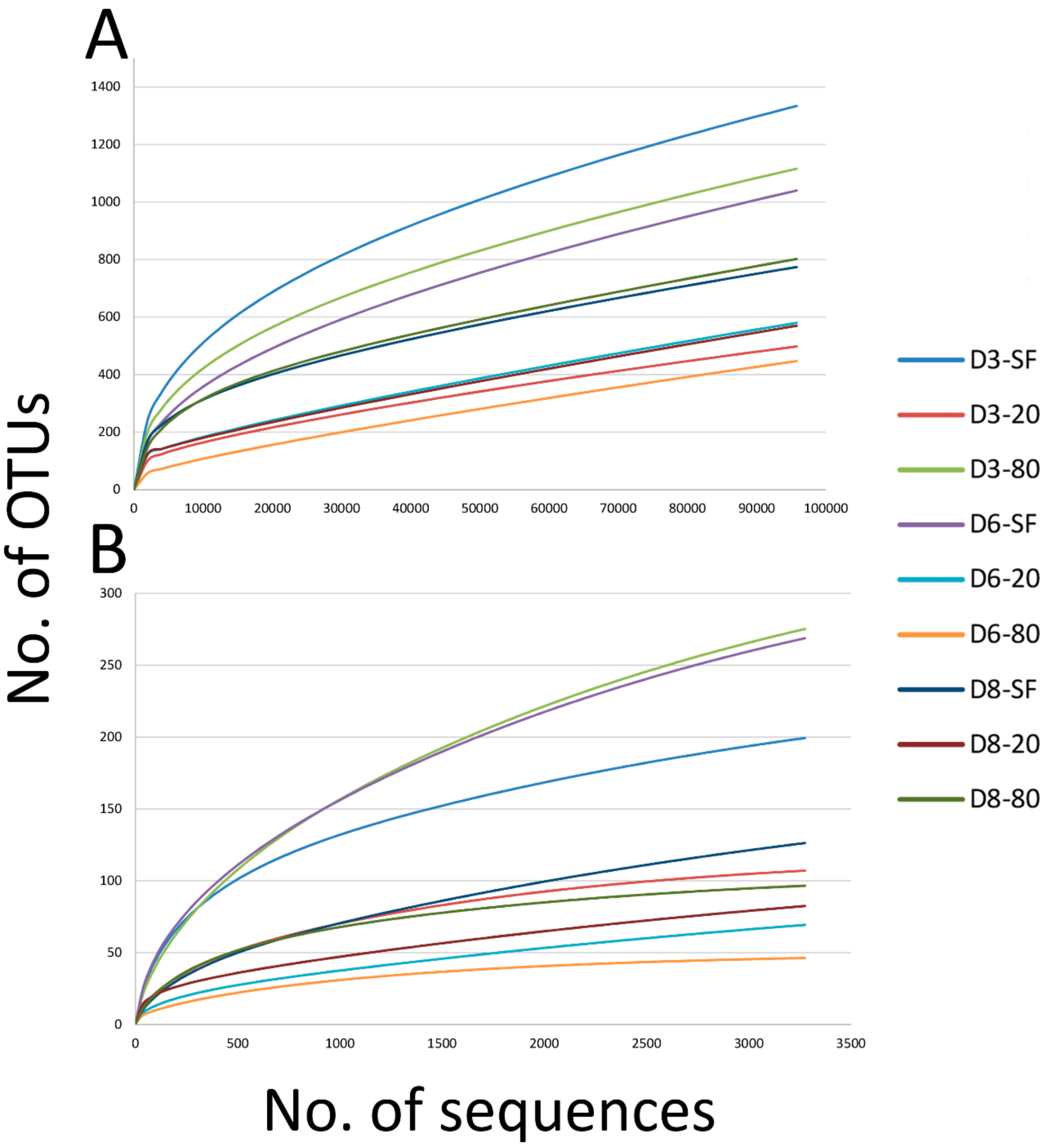

Supporting information S12. Rarefaction curves of (A) Bacterial and (B) Archaeal communities. Note differences between axis.

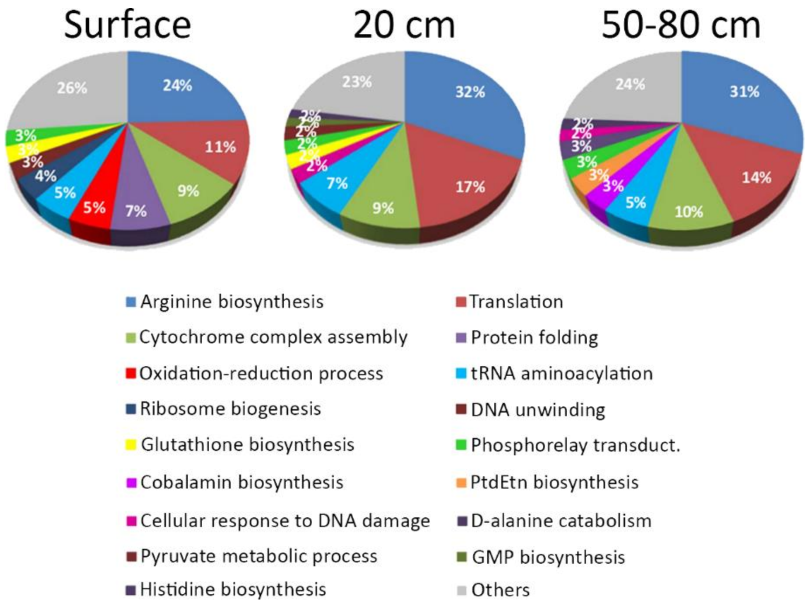

Supporting information S13. Circular charts showing the proportion of unique proteins grouped by main biological process categories identified by GO terms. 'Surface' group comprises D3-SF, D6-SF and D8-SF samples; '20 cm' group comprises D3-20, D6-20 and D8-20 samples; '50-80 cm' group comprises D3-80, D6-80, D8-80 and D10-50 samples.

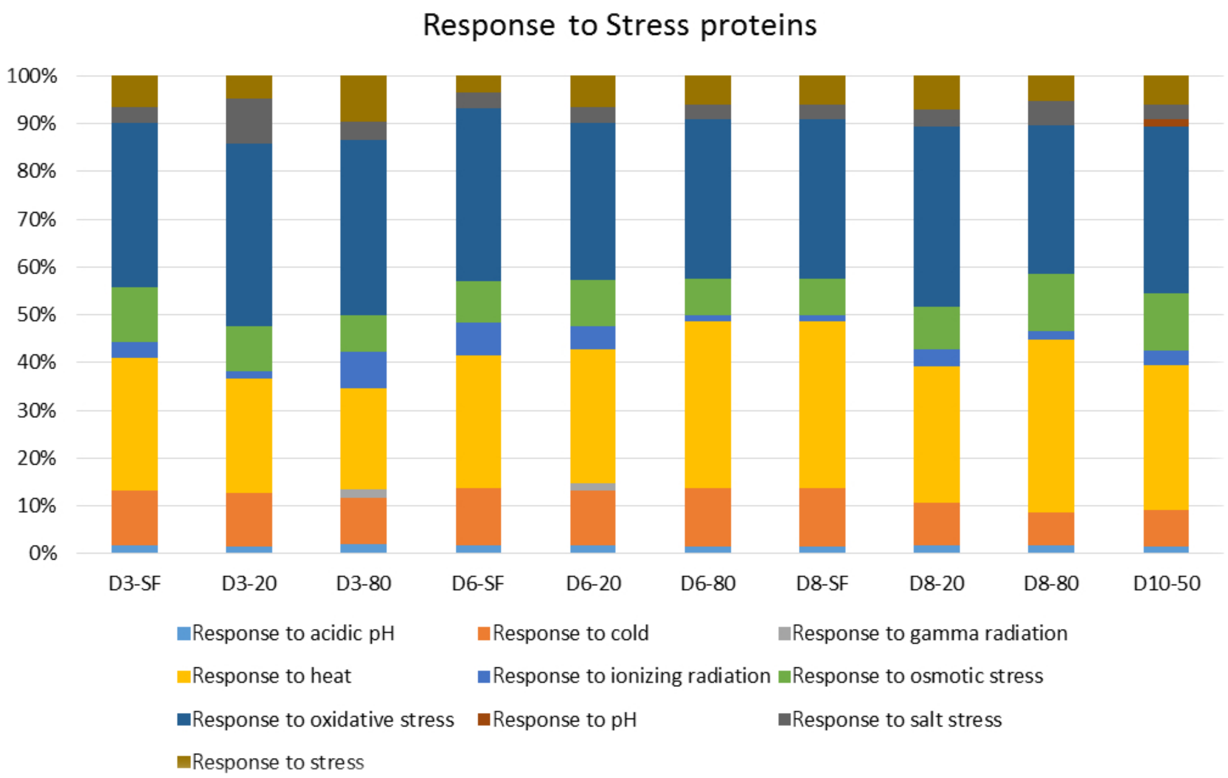

Supporting information S14. Distribution of stress resistance proteins across samples and depths.
